# Development of suspension cell culture model to mimic circulating tumor cells

**DOI:** 10.18632/oncotarget.23079

**Published:** 2017-12-07

**Authors:** Ji Young Park, Ae Lee Jeong, Hyun Jeong Joo, Sora Han, So-Hyun Kim, Hye-Youn Kim, Jong-Seok Lim, Myeong-Sok Lee, Hyung-Kyoon Choi, Young Yang

**Affiliations:** ^1^ Department of Biological Sciences, Sookmyung Women’s University, Seoul 04312, Republic of Korea; ^2^ College of Pharmacy, Chung-Ang University, Seoul 06974, Republic of Korea

**Keywords:** suspension cells, metastasis, lipidomic profile, metabolic profile

## Abstract

Circulating tumor cells (CTCs) are essential for the establishment of distant metastasis. Numerous studies have characterized CTCs as metastatic precursors; however, the molecular nature of CTCs has not been completely revealed yet due to the low number of CTCs in the blood stream. As an alternative approach, we developed a long-term suspension cell culture model using human breast cancer cell lines to mimic CTCs. We found that more than 40 passaged suspension cells acquired the ability to enhance metastasis like cancer stem cells. To identify molecular changes acquired during the suspension cell culture, we analyzed metabolic and lipidomic profiles as well as transcriptome in MDA-MB-468 suspension cells. Glutamate and leucine levels increased in suspension cells, and cholesterol synthesis pathway was altered. The inhibition of glutamate metabolic pathway decreased the proliferation of suspension cells compared to that of adherent cells. In the lipidomic profile, PC species containing long chain and polyunsaturated fatty acids increased in suspension cells and these species could be authentic and specific biomarkers for highly metastatic cancers. As this CTC-mimicking suspension cell culture model may easily apply to various types of cancer, we suggest this model as a great tool to develop therapeutic targets and drugs to eradicate metastatic cancer cells.

## INTRODUCTION

Epithelial mesenchymal transition (EMT) is the first step for tumor cells to leave their original site. As the dissemination of tumor cells mostly occurs through the blood stream after EMT, tumor cells that have been shed into the vasculature are known as circulating tumor cells (CTCs) [1]. On the other hand, cancer stem cells (CSCs) are known as self-renewal and tumor-initiating cells [2]. CSCs also travel into the blood stream as CTCs. Thus, CTCs with high metastatic potential may act as CSCs. However, the relationship between CSCs and CTCs remains unclear. CTCs express cytokeratins and do not have CD45, indicating that CTCs are not of hematopoietic origin but of epithelial origin [3]. Cytokeratin-negative CTCs are considered as CSCs or cells undergoing EMT [4]. Although CTCs have important prognostic and therapeutic implications and are useful for liquid biopsy, these cells are not easily detected because they exist in a very small amount. To overcome this hurdle, we cultured adherent tumor cells in suspension and continuously maintained in suspension to mimic CTCs or CSCs, and a comprehensive and comparative analysis on gene expression and metabolism was performed to characterize suspension cells.

Metabolism allows transformation of nutrients into energy, reducing power, and cellular building blocks necessary for life. Recently, metabolomics, which analyzes all observable metabolites in biological samples, has emerged as a new promising tool for identification of therapeutic targets and diagnostic and prognostic markers in cancer research [5]. Recent studies have described essential metabolomic pathways in breast cancer and characterized oncometabolites that drove tumor growth and progression [6–8]. Furthermore, in order to obtain information on cellular function of metabolites, analyses of metabolites coupled with genetic or lipidomic profiling have been performed [9, 10].

Biological membranes consist of different species of lipids, which modulate membrane-dependent cellular functions [11, 12]. Thus, alterations in lipid species influence biological processes through the change of lipid signal transduction pathways and are strongly correlated with cancer and other human diseases [13, 14]. Therefore, lipidomic analysis has been used to determine the functional relationship between lipid alterations and physiological function in cancer and various stem cells [15–18]. We analyzed metabolic and lipidomic profiles to identify molecular targets in CTC-mimicking suspension cells in order to eliminate CTCs, which could be metastasized.

## RESULTS

### Adherent and suspension breast cancer cells

Breast cancer can be classified into five subtypes based on gene expression profiling and immunohistochemical expression of estrogen receptor α (ERα), progesterone receptor (PR), and human epidermal growth factor receptor 2 (HER2): luminal A, luminal B, HER2, and basal- and normal-like [19–21]. We generated stable suspension cells with breast cancer cell lines representing each type: T47D (luminal A), ZR-75-1 (luminal B), SK-BR-3 (HER2), MDA-MB-231 (claudin-low), MDA-MB-468 and HCC1937 (basal-like). To generate suspension cells, cells were synchronized to mitotic phase by nocodazole treatment and round-shaped cells isolated by shaking were cultured with RPMI supplemented 10% FBS on an ultra-low attachment plate. This mitotic arrest makes adherent cells to round-shaped cells spontaneously and release of cell cycle makes round-shaped whole cells to face with simultaneous selection pressure at the same cell cycle. Given that MDA-MB-468 cells were isolated from a pleural effusion of a patient with metastatic adenocarcinoma of the breast, suspension MDA-MB-468 cells could behave like CTCs or CSCs having metastatic potential. Therefore, comparison between adherent and suspension MDA-MB-468 cells was preferentially performed by analyzing features of the transcriptome and metabolic and lipidomic profiles.

### Phenotypic/genotypic analysis of suspension MDA-MB-468 cells

**Figure 1:**
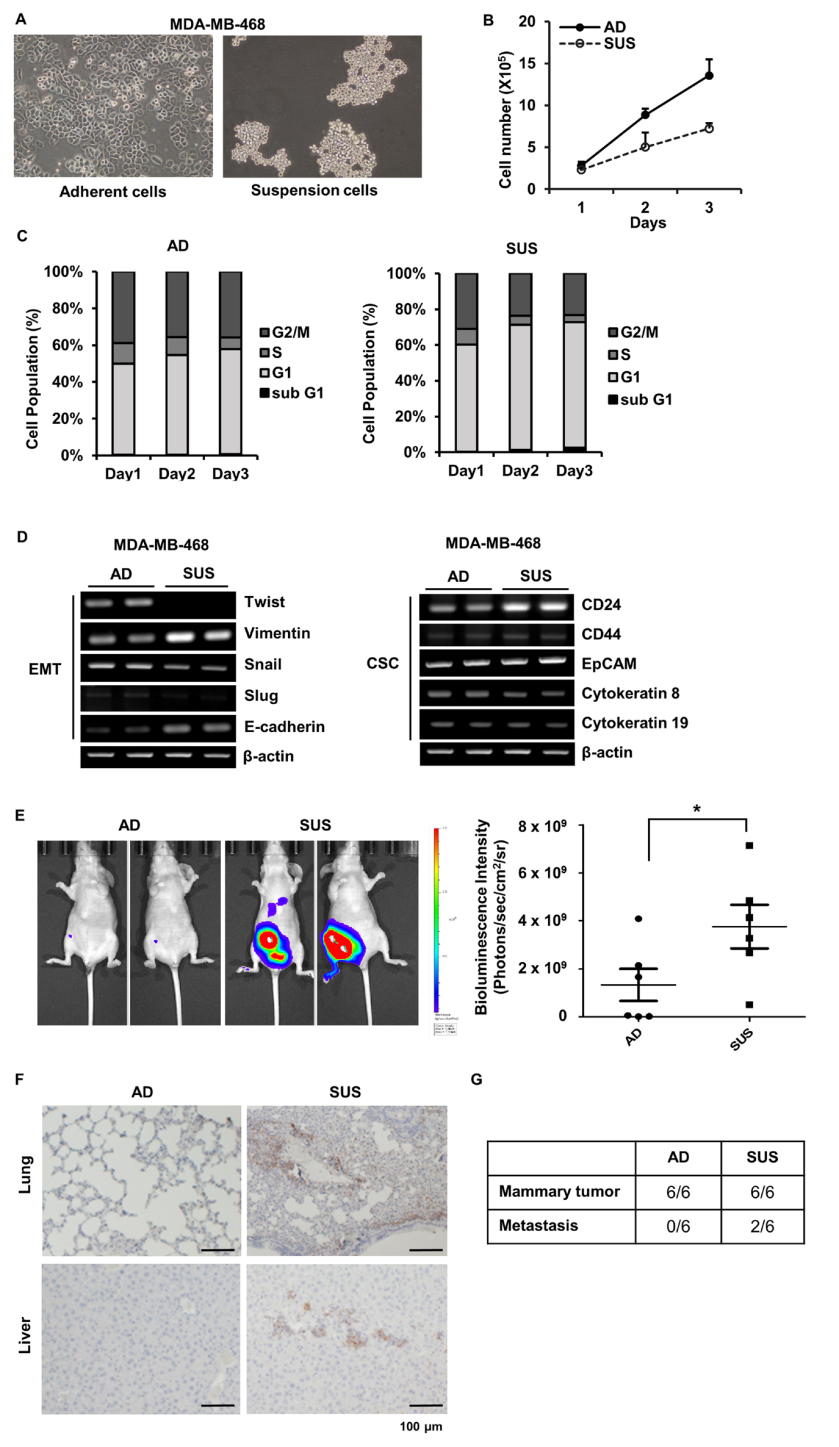
Phenotypic and genotypic analysis of adherent and suspension MDA-MB-468 cells (**A**) Adherent and suspension cells were photographed. (**B**) Number of adherent and suspension cells were directly counted at the indicated day. (**C**) Cell cycle of adherent and suspension cells was analyzed using flow cytometry at the indicated day. (**D**) Total RNAs were isolated, and EMT and CSCs marker genes were amplified using RT-PCR. (**E**) Stably expressing luciferase MDA-MB-468 adherent and suspension cells were injected into the mammary fat pad of athymic nu/nu mice and the *in vivo* bioluminescent signal was quantified using IVIS Lumina XRMS. Representative *in vivo* images of adherent or suspension cells injected mice and a dot plot comparing the bioluminescent signal in each group (mean ± SEM, *n* = 6) are shown. ^*^p < 0.05; two-tailed Mann Whitney *t*-test (**F**) Representative images of lung and liver tissue sections staining with vimentin in two groups of animals are shown (Scale ba*r* = 100 µm). (**G**) The number of mice showing mammary tumor formation and metastases were indicated. AD, adherent cells; SUS, suspension cells.

More than 40 passages cells were used to analyze characteristic of suspension cells. Suspension cells grew in grape-like clusters that floated in the medium (Figure 1A) and more slowly proliferated than adherent cells (Figure 1B). To find out a reason why suspension cells proliferate slowly, cell cycle analysis was performed. Suspension cells showed increase in G1 phase population and no significant apoptotic cells were detected in both cells (Figure 1C). As CTCs are considered CSCs or cells undergoing EMT, in order to determine the characteristics of the suspension MDA-MB-468 cells, CSC and EMT markers were analyzed using RT-PCR. Adherent and suspension cells expressed cytokeratin, indicating an epithelial origin. Suspension cells showed decreased level of Twist and increased level of E-cadherin and Vimentin. Breast CSCs usually show the CD44^+^/CD24^−/low^ phenotype, but suspension cells showed an increase in CD24 with no change in CD44 and epithelial cellular adhesion molecule (EpCAM) (Figure 1D). Since it is still unclear to define the phenotype of CTCs because of heterogeneity of CTCs [22] and EMT is dispensable for metastasis in some cancer [23], this intricate marker expression on suspension cells is conceivable.

Next, we performed orthotopic xenograft experiments in athymic nude mice using adherent and suspension cells expressing luciferase to determine whether suspension cells have more efficient metastatic potential than adherent cells. Bioluminescence intensity was significantly increased in mice injected into mammary fat pad with suspension cells than adherent cells (Figure 1E). Tumor metastasis was examined by vimentin staining at lung and liver tissue sections. Mice injected with suspension cells showed a strong vimentin staining in lung and liver (Figure 1F). In addition, tumor cells in blood were assessed by measuring the ratio of human DNA content to mouse DNA content in cells isolated from whole blood to determine level of CTCs [24, 25]. CTCs were observed in two among six mice injected with suspension cells, but no CTCs were detected in all six mice injected with adherent cells (Figure 1F). Metastases were only observed in mice having CTCs (Figure 1G). To further confirm metastatic ability of suspension cells, we determined level of lung colonization following injection of adherent or suspension cells directly into the lateral tail vein of female NOD-scid-gamma (NSG) mice. Number of metastatic nodules were similar between two cells (Supplementary Figure 1A), but analyses of lung histology showed that vimentin positive metastatic area formed by suspension cells were about 1.92-fold greater than that of adherent cells (Supplementary Figure 1B and 1C). These findings imply that suspension cells acquire higher metastatic ability than adherent cells.

### Metabolic profiling of MDA-MB-468 cells

**Figure 2:**
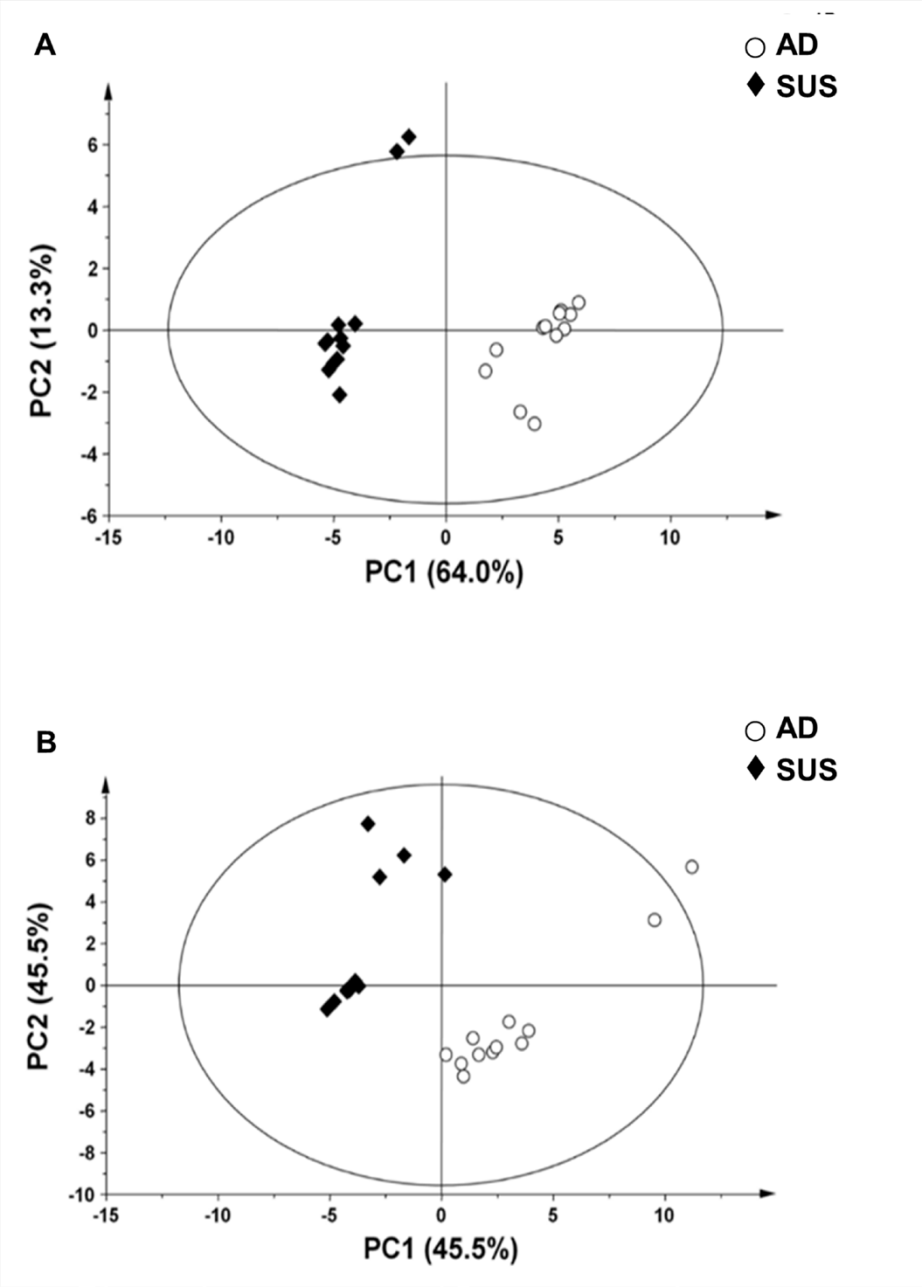
Principal component analysis (PCA) score plot derived from (**A**) GC-MS data and (**B**) nanoESI-MS data of adherent and suspension cells. PC1, principal component 1; PC2, principal component 2. AD, adherent cells; SUS, suspension cells.

In order to identify the molecular factors that contributed to the characteristics of suspension cells, metabolic, lipidomic, and trasnscriptome analyses were performed. GC-MS and nanoESI-MS were used to analyze the difference in metabolite profiles between adherent and suspension MDA-MB-468 cells. In order to evaluate whether the changes in metabolite profile were induced, the processed mass spectral data were subjected to PCA. The PCA score plot revealed a clear separation between adherent cells and suspension cells (Figure 2). These results implied that MDA-MB-468 cells underwent a transformation of their metabolic profile during cultivation in suspension culture system.

**Figure 3:**
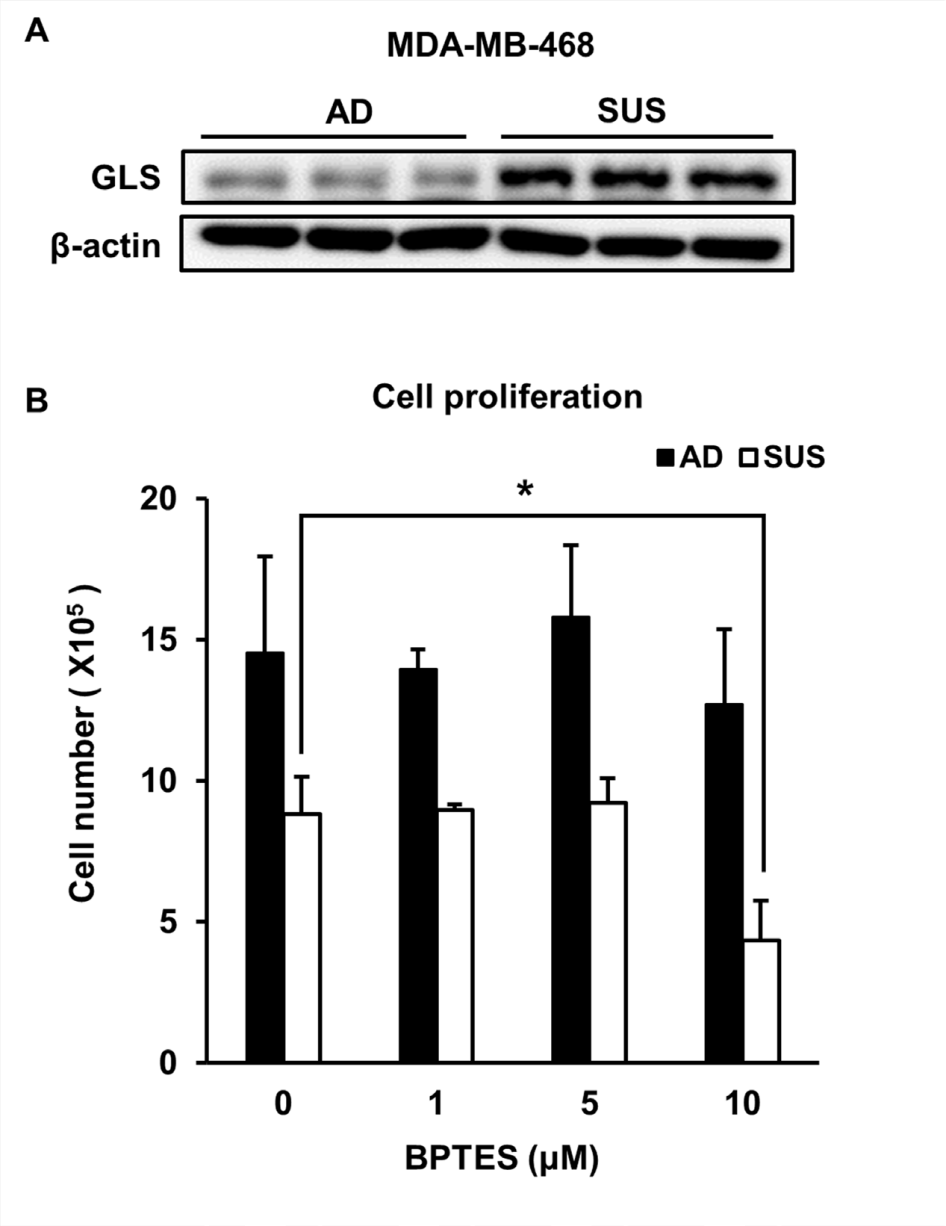
Suspension cells show an increase in GLS and are susceptible to treatment with GLS inhibitor **(A)** Levels of GLS were examined using immunoblot assay. (**B**) Adherent and suspension cells were plated at a density of 3 × 10^5^ cells per well and cultured for 3 days in the presence of BPTES. Viable cells were counted. ^*^p < 0.05, ^**^p < 0.01, ^***^p < 0.001; two-tailed Student’s *t*-test.

The levels of most metabolites derived from suspension cells were low compared to those derived from adherent cells (Table 1). In particular, amino acid levels, except glutamic acid and leucine, decreased in suspension cells. Glutamine to glutamate conversion is catalyzed by various enzymes, including glutaminase (GLS) [26–28]. Interestingly, suspension cells showed an increase in GLS level (Figure 3A). In order to determine whether the level of glutamate was a critical requirement for the proliferation of suspension cells, cells were treated with the GLS inhibitor, BPTES. The proliferation of suspension cells dramatically decreased after BPTES treatment, whereas adherent cells were not affected at that concentration (Figure 3B).

**Table 1:** Metabolic profiles of adherent and suspension MDA-MB-468 human breast cancer cells using GC-M

Compound^a^	RT	Mass fragment (m/z)^b^	Relative intensities^c^		TMS^d^
			Adherent	Suspension	
Alcohols					
Arabitol^***^	21.61	103, 305, 217, 307	0.91 ± 0.23	0.26 ± 0.10	3
Myo-inositol^***^	28.93	191, 217, 305, 318	39.38 ± 4.19	20.85 ± 3.82	6
Glucitol	26.25	103, 205, 217, 319	0.23 ± 0.04	0.27 ± 0.08	6
Amino acids					
Alanine	7.02	116, 133, 191, 218	6.04 ± 0.72	5.38 ± 0.99	2
Asparagine^***^	19.22	116, 130, 159, 261	1.39 ± 0.16	0.19 ± 0.05	2
Aspartic acid^***^	15.07	117, 130, 160, 245	2.73 ± 0.29	1.64 ± 0.35	2
Glutamic acid^***^	19.76	128, 156, 246, 348	9.28 ± 2.08	21.40 ± 8.45	3
Glycine^**^	7.48	59, 102, 176, 204	23.26 ± 7.07	14.06 ± 2.36	2
	12.02	133, 174, 248, 276			3
Hydroxyproline^***^	16.29	75, 103, 158, 260	1.98 ± 0.21	0.50 ± 0.17	2
Isoleucine	8.84	86, 130, 146, 188	7.73 ± 1.24	6.75 ± 1.38	1
Leucine^***^	8.32	86, 103, 146, 188	5.65 ± 1.06	10.52 ± 2.59	1
Methionine^***^	14.72	56, 61, 104, 221	2.35 ± 0.34	1.12 ± 0.43	1
Phenylalanine^***^	18.00	91, 120, 146, 222	4.80 ± 0.54	1.68 ± 0.28	1
Proline	8.78	70, 75, 103, 172	9.56 ± 2.07	7.31 ± 3.36	1
Pyroglutamic acid^***^	17.37	133, 156, 230, 258	24.05 ± 3.14	5.66 ± 1.78	2
Serine^***^	10.83	103, 116, 132, 234	9.82 ± 1.57	6.82 ± 1.22	2
Threonine^***^	11.76	117,130, 158, 219	6.76 ± 0.98	3.20 ± 0.61	2
Tyrosine^***^	25.34	179, 208, 219, 310	21.59 ± 2.09	7.37 ± 0.86	2
	26.36	100, 179, 218, 280			3
Valine^***^	6.75	72, 130, 146, 174	14.98 ± 2.47	8.68 ± 1.50	1
Organic acids					
Aminomalonic acid^***^	16.16	133, 174, 218, 320	2.08 ± 0.35	1.10 ± 0.27	3
Fumaric acid^***^	13.26	83, 133, 143, 245	1.19 ± 0.12	0.54 ± 0.11	2
2-Hydroxyglutaric acid^***^	18.74	129, 157, 247, 349	0.29 ± 0.03	0.09 ± 0.02	3
Lactic acid^***^	6.10	117, 133,191, 219	533.52 ± 69.31	353.18 ± 65.13	2
Malic acid^***^	16.66	133, 233, 245, 335	5.91 ± 0.67	2.91 ± 0.49	3
Fatty acids					
Palmitic acid	28.34	117, 129, 145, 313	0.23 ± 0.06	0.25 ± 0.06	1
Stearic acid	31.48	117, 129, 145, 341	0.22 ± 0.09	0.26 ± 0.09	1
Sugars					
Glucose	25.47	103, 191, 204, 217	21.16 ± 2.89	23.46 ± 7.23	5
	25.52	160, 205, 217, 319			5(1MEOX)
	27.13	103, 191, 204, 217			5
Glucose-6-phosphate	32.47	129, 299, 357, 387	6.09 ± 1.47	6.90 ± 1.34	6
	33.31	204, 299, 315, 387			6
Glyceric acid^***^	12.68	103, 133, 189, 292	1.24 ± 0.25	0.81 ± 0.19	3
Mannaose-6-phosphate^***^	32.30	217, 299, 315, 387	2.99 ± 0.60	6.64 ± 1.35	6
Ribose^***^	21.00	103, 189, 217, 307	1.92 ± 0.27	3.05 ± 0.57	3(1MEOX)
	25.25	103, 217, 277, 307			4(1MEOX)
Purine Guanine^***^	29.58	99, 264, 352, 367	ND	3.34 ± 1.84	3
Pyrimidine Uridine^***^	34.31	103, 169, 217, 259	9.70 ± 1.93	2.02 ± 0.48	3

^a^Asterisk-superscripts denote statistical significant differences (Student’s *t*-test; ^**^p < 0.01; ^***^p < 0.001) among two groups, adherent and suspension MDA-MB-468 human breast cancer cells.

^b^Bold-faced numbers in mass fragment ion mean base peak.

^c^Values are presented as mean ± standard deviation of technical duplicates on 6 individual biological replicates; ND, not detected.

^d^Numbers indicate the number of trimethylsilylation (TMS) incorporated upon derivatization; MEOX, methoxyaminated derivative.

### Lipid profiling of MDA-MB-468 cells

The representative nanoESI-MS spectra of lipids from a pooled lipid extract sample of adherent and suspension cells are illustrated in Supplementary Figure 2. In total, ten phosphatidylcholine (PC) species and one phosphatidylethanolamine (PE) species were detected in positive ion mode, while three ceramide (CER) species, seven PE species, three phosphatidylglycerol (PG) species, thirteen phosphatidylserine (PS) species, and eight phosphatidylinositol (PI) species were detected in negative ion mode. PE (P-16:0/20:4) was detected in both ion modes (Supplementary Table 1).

**Figure 4:**
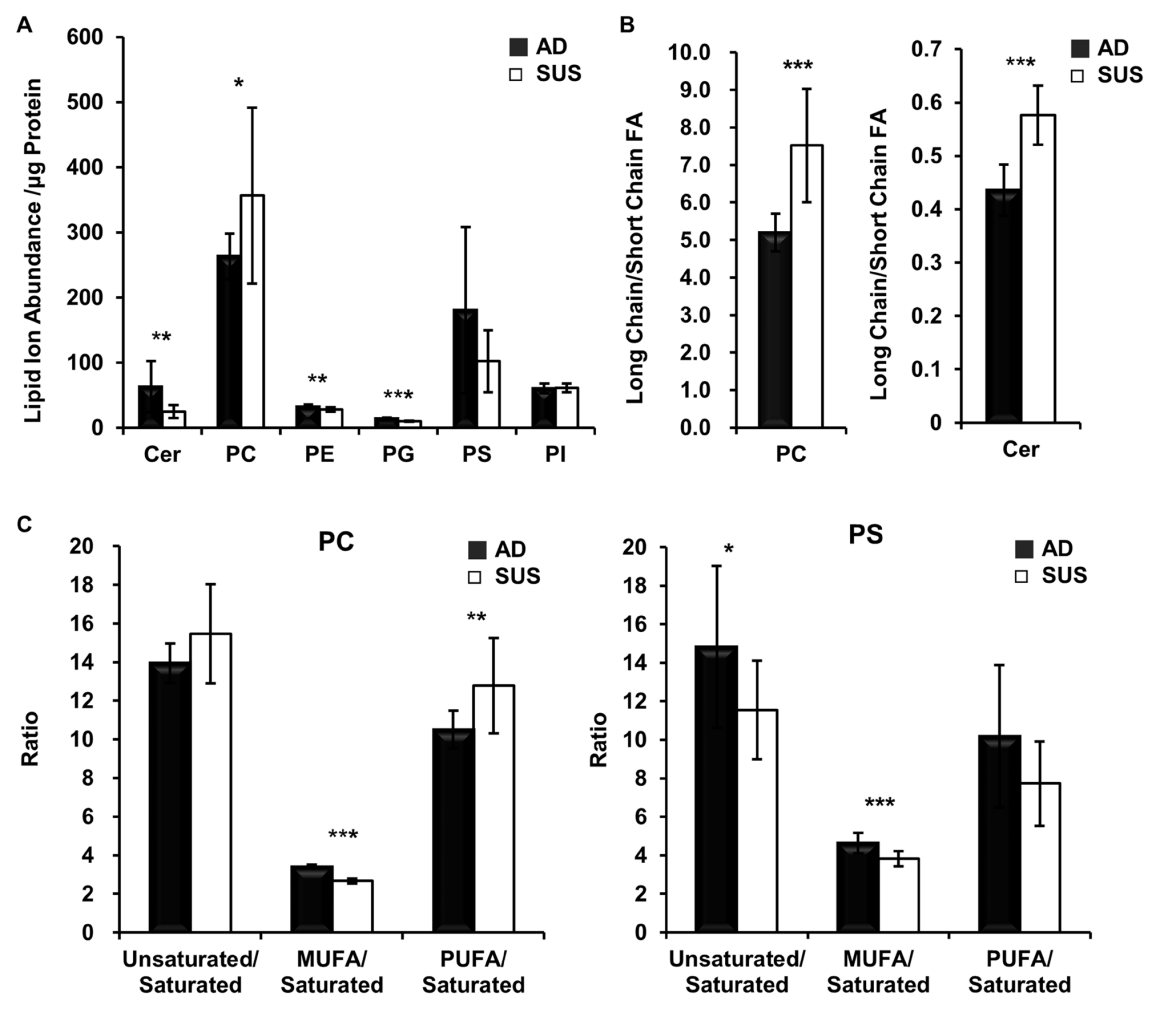
Suspension cells show an increase in PCs that have long chain and PUFA **(A)** Comparison of the total normalized summed ion abundances for each lipid class. (**B**) Ratio of the total normalized abundances of long chain versus short chain fatty acids. Fatty acids more than C18 are considered as long chain fatty acids. (**C**) Ratio of the total normalized abundances of unsaturated versus saturated of fatty acids. PUFA have more than two unsaturated bonds in a fatty acid.

In order to investigate the changes in lipid levels of suspension cells, the fold changes in adherent and suspension cells were estimated (Table 2) and total amount of each lipid species was obtained by summed ion abundances (Figure 4A). The levels of CER, PE and PG were lower in suspension cells than those in adherent cells, and only PC levels increased and each PC species varied. Next, relative ratio of phospholipid containing long chain and short chain fatty acids (less than C18) was determined. PC and Cer containing long chain fatty acids increased in the suspension cells (Figure 4B). At a ratio of saturated and unsaturated fatty acids, PCs have more poly unsaturated fatty acids (PUFA), but PSs less PUFA (Figure 4C).

**Table 2:** Fold changes (suspension/adherent) and p-values of compounds identified from MDA-MB-468 human breast cancer cells

Compounds^a^	Fold change	*p*-value^b^
Cer (d18:1/16:0)	0.33	2.07E-03
Cer (d18:1/17:0)	0.56	n.s.
Cer (d18:1/18:0)	0.48	6.18E-03
PC (14:0/16:0)	0.68	1.64E-06
PC (16:0/16:0)	1.70	1.85E-07
PC (16:0/16:1)	0.83	3.83E-03
PC (16:0/18:1)	0.98	n.s.
PC (16:1/18:1)	1.20	n.s.
PC (18:0/22:5)	1.48	4.42E-03
PC (18:1/18:1)	1.51	1.34E-02
PC (18:1/18:2)	1.69	3.17E-03
PC (18:1/20:4)	1.36	n.s.
PC (18:1/20:5)	1.76	2.46E-02
PE (P-16:0/20:4)	0.93	n.s.
PE (18:0/18:1)	0.74	1.59E-04
PE (18:0/20:4)	1.89	8.00E-11
PE (18:0/22:5; 18:1/22:4)	1.45	7.47E-06
PE (18:1/18:1)	0.49	9.97E-14
PE (18:1/20:4)	1.01	n.s.
PG (16:0/18:1)	0.79	1.20E-05
PG (18:0/18:1)	0.80	n.s.
PG (18:1/18:1)	0.50	8.14E-09
PS (16:0/18:0)	0.70	n.s.
PS (16:0/18:1; 16:1/18:0)	0.57	n.s.
PS (16:1/18:1)	0.47	n.s.
PS (18:0/18:0)	0.77	9.58E-03
PS (18:0/18:1)	0.60	1.62E-03
PS (18:0/20:1)	1.03	n.s.
PS (18:0/20:3)	2.44	3.45E-09
PS (18:0/20:4)	3.73	4.97E-03
PS (18:0/22:5)	0.67	1.97E-06
PS (18:0/22:6)	0.97	n.s.
PS (18:1/18:1)	0.42	3.46E-02
PS (18:1/18:2)	0.78	n.s.
PI (16:0/18:1)	0.57	7.28E-08
PI (16:0/20:4)	1.41	1.63E-07
PI (16:1/18:1)	0.83	n.s.
PI (18:0/20:3)	1.29	5.07E-07
PI (18:0/20:4)	1.58	2.00E-11
PI (18:1/18:1)	0.43	2.36E-15
PI (18:1/18:2)	1.12	1.07E-02
PI (18:1/20:4)	0.90	2.75E-02

^a^Cer, ceramide; PC, phosphatidylcholine; PE, phosphatidylethanolamine; PG, phosphatidylglycerol; PS, phosphatidylserine; PI, phosphatidylinositol.

^b^n.s., not statistically significant (*p* < 0.05).

Gene ontology (GO) functional enrichment analysis of differentially expressed genes

**Figure 5:**
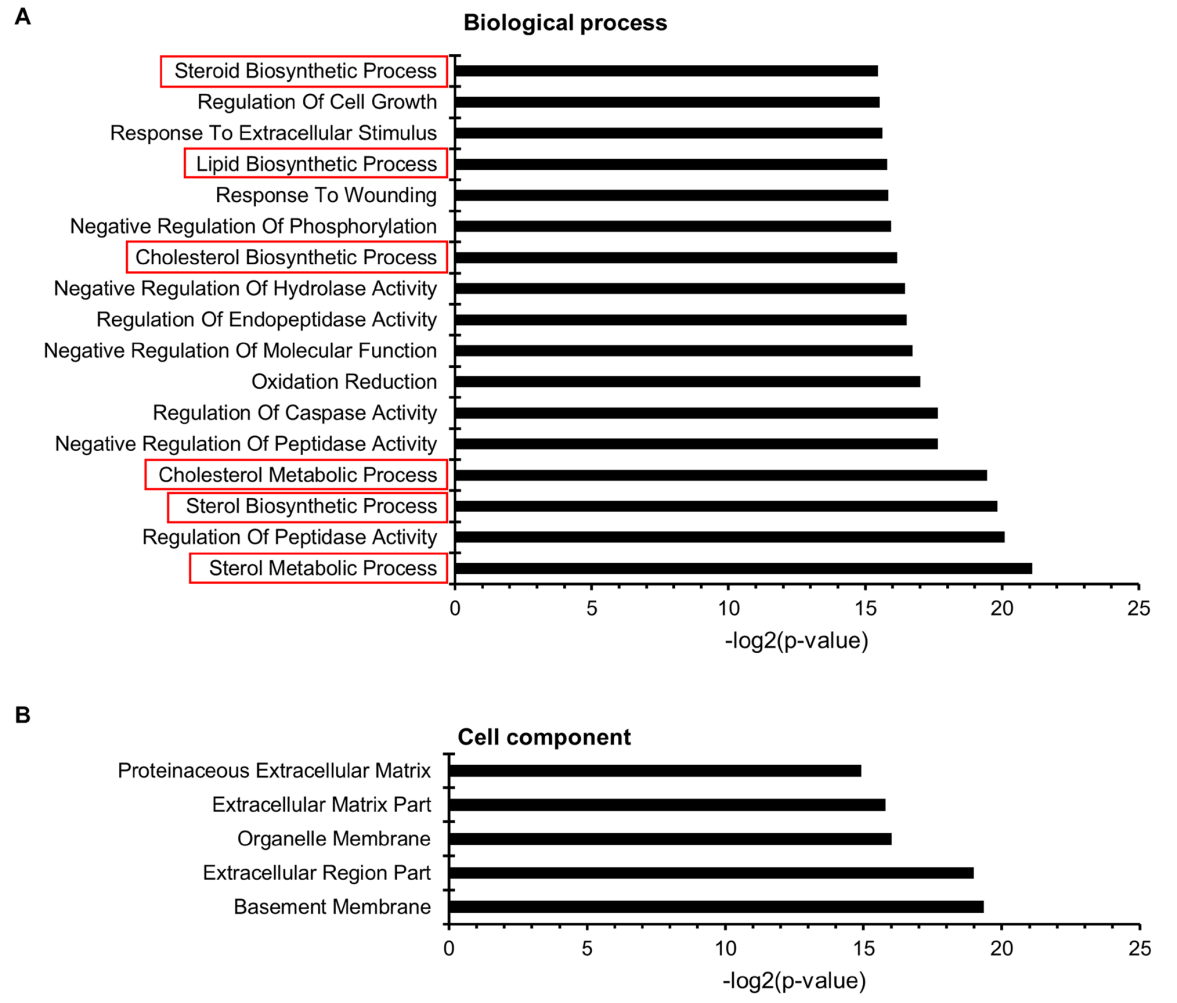
Microarray gene ontology (GO) classification The x-axis indicates the likelihood [−log_2_ (*p*-value)] in a category, and the y-axis indicates the different subcategories of (**A**) biological process and (**B**) cell component. The GO terms related to lipid metabolism are represented by red boxes.

In order to link metabolic and lipidomic profile changes with transcriptome data, the difference in gene expression patterns between adherent and suspension cells was analyzed. RNA-seq analysis revealed that 2,873 genes were differentially expressed (*p* < 0.05, False Discovery Rate (FDR) < 0.01), with greater than 2-fold change. Among these genes, 1,466 genes were significantly up-regulated, while 1,407 were down-regulated in suspension cells compared to that in adherent cells. To determine the metabolic processes that differed between adherent and suspension cells, the three GO categories, namely biological process, cell component, and metabolic function, were explored using DAVID bioinformatics tools, and biological functions with an FDR value greater than 0.05 were considered significant. In total, 1,268 differentially expressed genes were annotated in 22 GO functional groups, including 17 groups in biological process, 5 groups in cellular component, and no group in molecular function (Figure 5). In the biological process category, the most important enriched terms were related to metabolic and biosynthetic processes of sterols, steroids, and lipids. Within the cellular component category, the GO terms with the highest level of significance were basement membrane, extracellular region, and matrix part. To analyze the functional relationship between lipid profiling and RNA-seq results, we focused on 6 GO terms for the biological process related to lipid and steroid metabolism (Table 3).

**Table 3:** List of genes related to lipid and sterol metabolism

GOTerm_BP	*p*-value	Count	Genes
Sterol Metabolic Process	4.48E-07	35	CYB5R3, LDLR, MVD, HMGCR, CYP51A1, HMGCS1, APOC1, LSS, ABCA1, C14ORF1, SCARF1, FDFT1, CYP39A1, INSIG2, DHCR7, INSIG1, PCSK9, SCARB1, CAT, NSDHL, DHCR24, SOAT1, EBP, FDPS, FDXR, SIGMAR1, ABCG1, CEL, CYP7B1, APOL1, CYP27A1, SQLE, MVK, IDI1, SC5DL
Sterol Biosynthetic Process	1.08E-06	18	CYB5R3, EBP, MVD, HMGCR, CYP51A1, HMGCS1, FDPS, LSS, C14ORF1, SIGMAR1, FDFT1, SQLE, DHCR7, MVK, IDI1, SC5DL, DHCR24, NSDHL
Cholesterol Metabolic Process	1.39E-06	32	CYB5R3, MVD, LDLR, HMGCR, CYP51A1, HMGCS1, APOC1, LSS, ABCA1, SCARF1, FDFT1, CYP39A1, INSIG2, DHCR7, INSIG1, PCSK9, SCARB1, CAT, NSDHL, DHCR24, SOAT1, EBP, FDPS, FDXR, ABCG1, CEL, CYP7B1, APOL1, CYP27A1, SQLE, MVK, IDI1
Cholesterol Biosynthetic Process	1.36E-05	14	CYB5R3, EBP, MVD, HMGCR, CYP51A1, FDPS, HMGCS1, LSS, FDFT1, DHCR7, MVK, IDI1, DHCR24, NSDHL
Lipid Biosynthetic Process	1.75E-05	74	ALDH8A1, CYB5R3, GBGT1, HMGCR, EDN1, LSS, ACSS2, C14ORF1, FDFT1, ELOVL1, PECR, ST6GALNAC6, STARD5, CYP39A1, PTGIS, ANG, ELOVL4, ELOVL3, AGPAT9, QKI, ELOVL7, SCD5, PCYT2, FGF2, PIGA, DHCR24, SPTLC3, PIGU, PIGS, CERCAM, SIGMAR1, PNPLA3, TECR, CHPT1, LPCAT4, PLAUR, SQLE, C5ORF4, PLA2G6, MVK, KGFLP1, C5DL, HSD17B11, MVD, CYP51A1, HSD3B7, CHKB, HMGCS1, MIF, ISYNA1, TPI1, CYP27B1, DHCR7, FASN, ETNK2, SCARB1, ACSL3, NSDHL, CPT1B, EBP, MOGAT2, A4GALT, FADS1, SCD, FDXR, FDPS, ACLY, CYP7B1, GGT5, P2RX7, ISPD, MBOAT2, ALOX5, IDI1, MGST2
Steroid Biosynthetic Process	2.23E-05	28	CYB5R3, HSD17B11, MVD, HMGCR, CYP51A1, HSD3B7, HMGCS1, LSS, C14ORF1, FDFT1, STARD5, CYP39A1, CYP27B1, DHCR7, SCARB1, FGF2, NSDHL, DHCR24, EBP, FDPS, FDXR, SIGMAR1, TECR, CYP7B1, SQLE, MVK, IDI1, SC5DL

### Genes related to lipid metabolism

**Figure 6:**
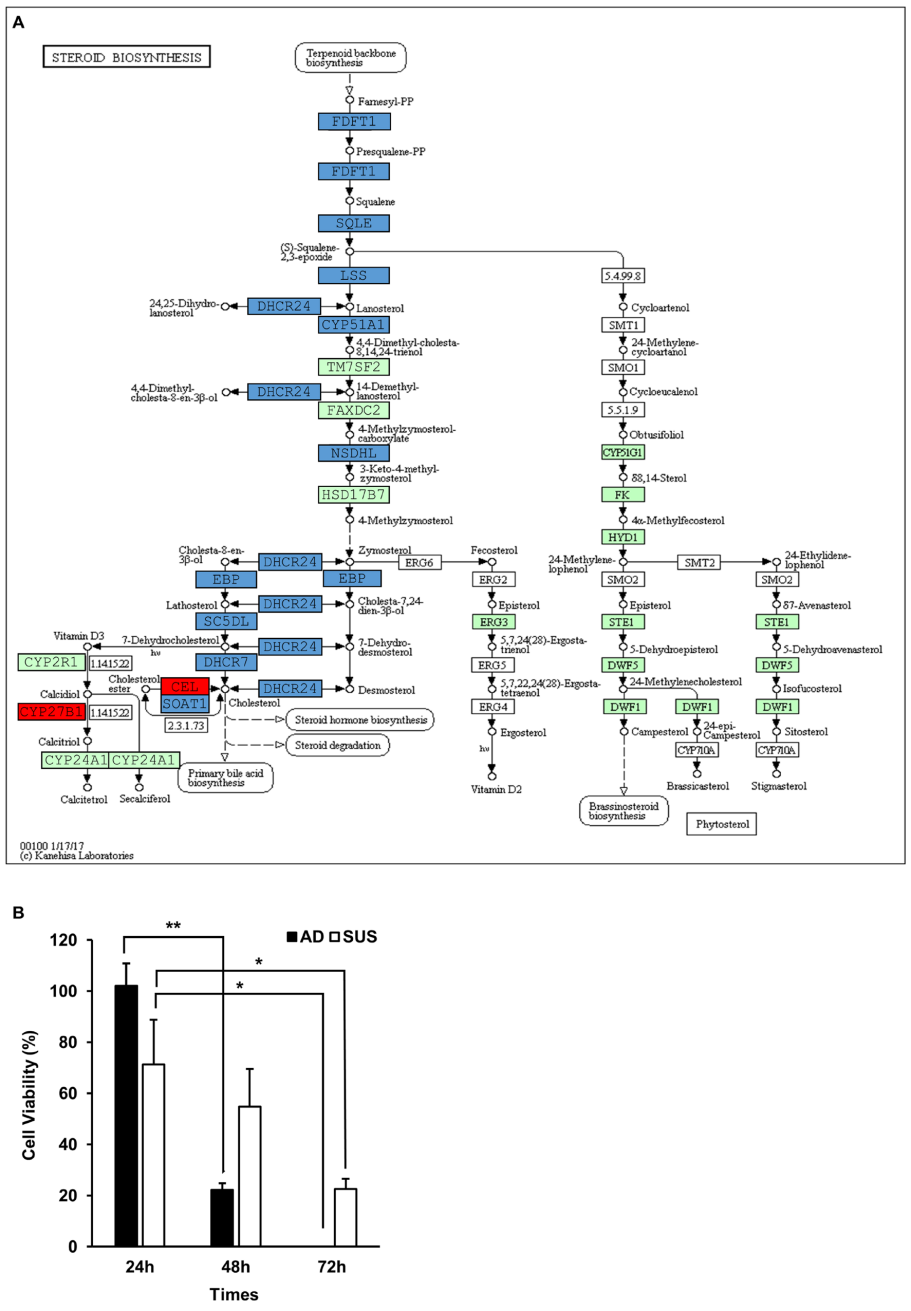
Viability of adherent cells was highly decreased by inhibition of cholesterol pathway (**A**) The significantly enriched genes in the steroid biosynthesis signal pathway. Red boxes indicate the significantly increased genes, and blue boxes indicate the significantly decreased genes. (**B**) Viability of adherent cells was highly decreased by simvastatin treatment. Cells were plated into 6-well plates at a density of 3 × 10^5^ cells per well and treated with 10 μM simvastatin. Viability of cells was assessed by trypan blue exclusion test at the indicated hours. ^*^p < 0.05, ^**^p < 0.01, ^***^p < 0.001; two-tailed Student’s *t*-test.

To gain further insight into the genes related to lipid metabolism in suspension cells, we extracted genes from 6 GO terms obtained by the GO enrichment analysis (Table 3). We found that 88 significantly differentially expressed genes were involved in lipid metabolism (Tables 4 and 5). For a detailed analysis, Kyoto Encyclopedia of Genes and Genomes (KEGG) analysis as a pathway-based analysis was performed, and showed that 12 significantly differentially expressed genes (*p* < 0.01, FDR < 0.05) were enriched in the steroid biosynthesis signal pathway (Figure 6A). This finding prompted us to examine whether the inhibition of cholesterol synthetic pathway was more susceptible to growth of adherent cells than that of suspension cells. Both cells were treated with simvastatin, HMG-CoA reductase inhibitor, to block cholesterol synthesis. As expected, the growth of adherent cells was more seriously damaged than that of suspension cells (Figure 6B).

**Table 4:** The significantly up-regulated lipid metabolism-related genes in the suspension MDA-MB-468 cells

Gene	Description	log_2_(fold_change)
ALDH8A1	Aldehyde Dehydrogenase 8 Family Member A1	3.8634
SCARF1	Scavenger Receptor Class F Member 1	3.05874
P2RX7	Purinergic Receptor P2X 7	2.76073
ABCG1	ATP Binding Cassette Subfamily G Member 1	2.44505
CYP27B1	Cytochrome P450 Family 27 Subfamily B Member 1	2.23211
MOGAT2	Monoacylglycerol O-Acyltransferase 2	2.14329
EDN1	Endothelin 1	1.96464
CEL	Carboxyl Ester Lipase	1.75736
CYP39A1	Cytochrome P450 Family 39 Subfamily A Member 1	1.67679
GPAT3	Glycerol-3-Phosphate Acyltransferase 3	1.52103
PIGA	Phosphatidylinositol Glycan Anchor Biosynthesis Class A	1.44996
INSIG2	Insulin Induced Gene 2	1.37597
PLA2G6	Phospholipase A2 Group VI	1.32606
ABCA1	ATP Binding Cassette Subfamily A Member 1	1.30521
STARD5	StAR Related Lipid Transfer Domain Containing 5	1.23634
FGF7P6	Fibroblast Growth Factor 7 Pseudogene 6	1.23373
QKI	KH Domain Containing RNA Binding	1.07864
SPTLC3	Serine Palmitoyltransferase Long Chain Base Subunit 3	1.0068
ANG	Angiogenin	#N/A
CHKB	Choline Kinase Beta	#N/A
CPT1B	Carnitine Palmitoyltransferase 1B	#N/A

**Table 5:** The significantly down-regulated lipid metabolism-related genes in the suspension MDA-MB-468 cells

Gene	Description	log_2_(fold_change)
PTGIS	Prostaglandin I2 (Prostacyclin) Synthase	–4.38964
APOC1	Apolipoprotein C1	–4.26849
APOL1	Apolipoprotein L1	–4.14778
PCSK9	Proprotein Convertase Subtilisin/Kexin Type 9	–3.98917
A4GALT	Alpha 1,4-Galactosyltransferase	–3.13062
FAXDC2	Fatty Acid Hydroxylase Domain Containing 2	–2.94314
PNPLA3	Patatin Like Phospholipase Domain Containing 3	–2.94113
SCD	Stearoyl-CoA Desaturase	–2.85593
FGF2	Fibroblast Growth Factor 2	–2.84993
DHCR7	7-Dehydrocholesterol Reductase	–2.66095
ETNK2	Ethanolamine Kinase 2	–2.61412
ELOVL4	ELOVL Fatty Acid Elongase 4	–2.47722
FASN	Fatty Acid Synthase	–2.44083
FADS1	Fatty Acid Desaturase 1	–2.19383
INSIG1	Insulin Induced Gene 1	–2.19298
EBP	Emopamil Binding Protein (Sterol Isomerase)	–2.18836
MVD	Mevalonate Diphosphate Decarboxylase	–2.13654
ELOVL3	ELOVL Fatty Acid Elongase 3	–2.03057
MVK	Mevalonate Kinase	–2.02055
LSS	Lanosterol Synthase (2,3-Oxidosqualene-Lanosterol Cyclase)	–1.91218
IDI1	Isopentenyl-Diphosphate Delta Isomerase 1	–1.88897
FDPS	Farnesyl Diphosphate Synthase	–1.84979
LDLR	Low Density Lipoprotein Receptor	–1.82226
C14ORF1	Chromosome 14 Open Reading Frame 1	–1.73982
HSD17B11	Hydroxysteroid 17-Beta Dehydrogenase 11	–1.72799
CYP27A1	Cytochrome P450 Family 27 Subfamily A Member 1	–1.71884
FDXR	Ferredoxin Reductase	–1.70033
FDFT1	Farnesyl-Diphosphate Farnesyltransferase 1	–1.68175
GBGT1	Globoside Alpha-1,3-N-Acetylgalactosaminyltransferase 1	–1.65937
ST6GALNAC6	ST6 N-Acetylgalactosaminide Alpha-2,6-Sialyltransferase 6	–1.63569
SOAT1	Sterol O-Acyltransferase 1	–1.62789
GGT5	Gamma-Glutamyltransferase 5	–1.6126
ISPD	Isoprenoid Synthase Domain Containing	–1.61245
TECR	Trans-2,3-Enoyl-CoA Reductase	–1.60378
ISYNA1	Inositol-3-Phosphate Synthase 1	–1.57524
DHCR24	24-Dehydrocholesterol Reductase	–1.56679
LPCAT4	Lysophosphatidylcholine Acyltransferase 4	–1.5494
MGST2	Microsomal Glutathione S-Transferase 2	–1.5233
PECR	Peroxisomal Trans-2-Enoyl-CoA Reductase	–1.4498
ACLY	ATP Citrate Lyase	–1.43626
SCD5	Stearoyl-CoA Desaturase 5	–1.4233
NSDHL	NAD(P) Dependent Steroid Dehydrogenase-Like	–1.42082
CERCAM	Cerebral Endothelial Cell Adhesion Molecule	–1.41546
CYP51A1	Cytochrome P450 Family 51 Subfamily A Member 1	–1.3948
ELOVL7	ELOVL Fatty Acid Elongase 7	–1.38885
SQLE	Squalene Epoxidase	–1.37995
ACSS2	Acyl-CoA Synthetase Short-Chain Family Member 2	–1.35018
MIF	Macrophage Migration Inhibitory Factor (Glycosylation-Inhibiting Factor)	–1.33722
CYP7B1	Cytochrome P450 Family 7 Subfamily B Member 1	–1.23914
ALOX5	Arachidonate 5-Lipoxygenase	–1.2341
PLAUR	Plasminogen Activator, Urokinase Receptor	–1.23365
TPI1	Triosephosphate Isomerase 1	–1.20651
PIGU	Phosphatidylinositol Glycan Anchor Biosynthesis Class U	–1.2031
CYB5R3	Cytochrome B5 Reductase 3	–1.19764
CHPT1	Choline Phosphotransferase 1	–1.19349
SC5DL	Sterol-C5-Desaturase	–1.19039
ACSL3	Acyl-CoA Synthetase Long-Chain Family Member 3	–1.18695
PIGS	Phosphatidylinositol Glycan Anchor Biosynthesis Class S	–1.11575
PCYT2	Phosphate Cytidylyltransferase 2, Ethanolamine	–1.08604
SCARB1	Scavenger Receptor Class B Member 1	–1.0724
HMGCR	3-Hydroxy-3-Methylglutaryl-CoA Reductase	–1.04385
HMGCS1	3-Hydroxy-3-Methylglutaryl-CoA Synthase 1	–1.03708
MBOAT2	Membrane Bound O-Acyltransferase Domain Containing 2	–1.02974
HSD3B7	Hydroxy-Delta-5-Steroid Dehydrogenase, 3 Beta- And Steroid Delta-Isomerase 7	–1.02875
ELOVL1	ELOVL Fatty Acid Elongase 1	–1.02678
CAT	Catalase	–1.01705
SIGMAR1	Sigma Non-Opioid Intracellular Receptor 1	–1.00092

In addition to the change of genes related with cholesterol biosynthesis, levels of PC, PE, PS, and PI species varied depending on each species. For example, levels of PE species with two C18 fatty acyl chains significantly decreased, while levels of PE species with C18 and polyunsaturated fatty acyl chains, including C20:4, C22:4, and C22:5, significantly increased. Transcriptome data showed that levels of stearoyl-CoA desaturase-1 and -5 (SCD1 and SCD5), which form a mixture of 16:1 and 18:1 unsaturated fatty acids, decreased. Levels of elongation of very long chain fatty acids (ELOVL 1/3/7), which allows the addition of two carbons to the chain of long- and very long-chain fatty acids, was reduced. Phospholipase A2 Group VI (PLA2G6) catalyzes the release of fatty acids from phospholipids and plays a role in phospholipid remodeling. Collectively, the changes in SCD, ELOVL, and PLA2G6 expression contributed to the change of phospholipid profile in suspension MDA-MB-468 cells.

### Comparison of transcriptome data with published public databases of CTCs

**Figure 7:**
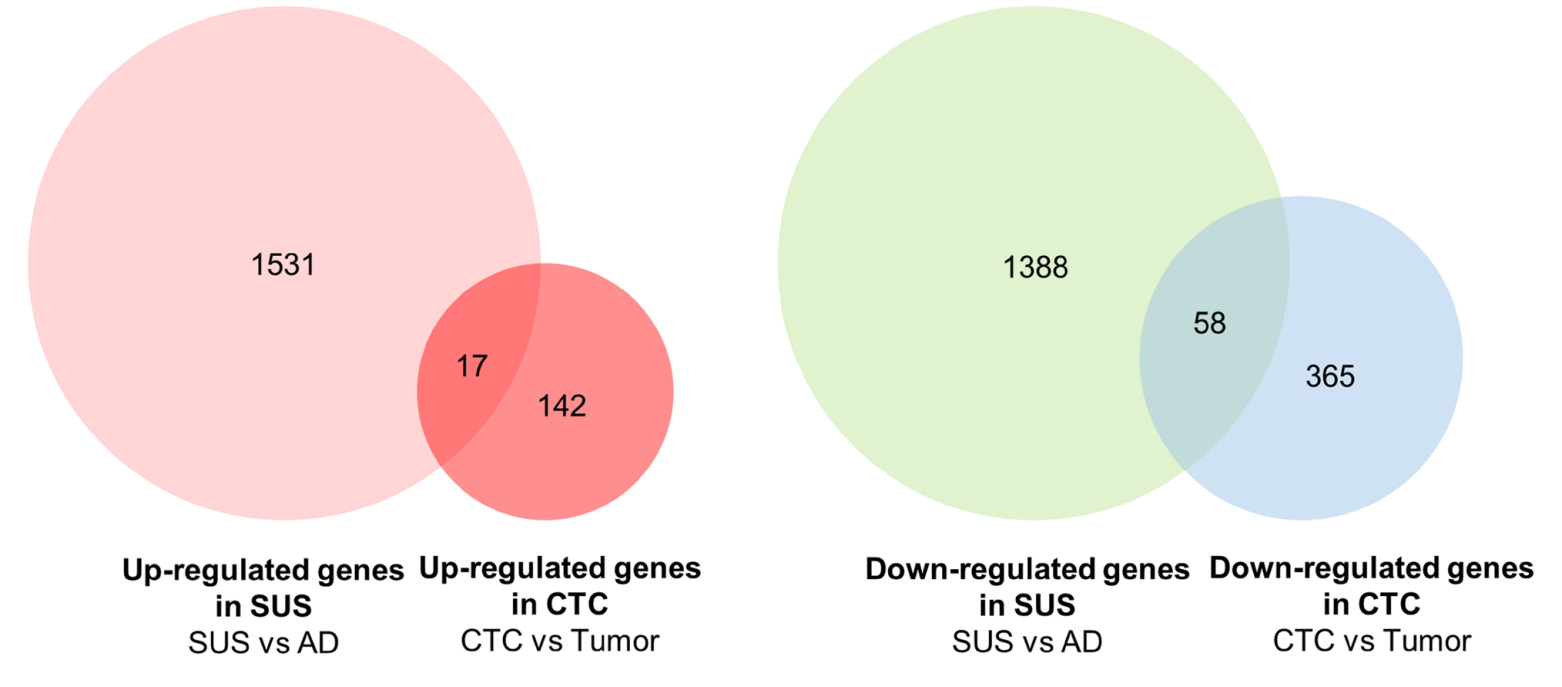
Venn diagram showing comparison of up- and down-regulated genes between MDA-MB-468 suspension cells and CTCs vs primary breast tumors Genes showing *p*-value < 0.05 and log_2_ fold change > 1 in suspension cells were compared with up- and down-regulated genes in CTCs vs primary breast tumors.

Next, we have compared our gene lists with published CTCs datasets. Most of datasets were obtained from single CTCs vs CTCs-cluster or CTCs vs leukocytes [22, 29, 30]. We have found one human dataset obtained from CTCs vs primary tumor tissues in the same breast cancer patient. To find out commonly expressed genes between suspension cells and CTCs obtained from breast cancer patients, the up- and down-regulated genes in suspension vs adherent cells were compared with up- and down-regulated genes in CTCs vs primary tumor tissues, respectively [31]. Among up-regulated genes in CTCs, 10.7% of genes are commonly increased in suspension cells and 13.7% of genes among down-regulated genes in CTCs are also decreased in suspension cells (Figure 7 and Supplementary Table 2). Functional annotation analysis with DAVID bioinformatics tools (http://david.abcc.ncifcrf.gov) were performed with 75 commonly regulated genes. It reveals that 11 genes including ATP5G1, ATP5G3, ATP6V1F, NDUFS5, ALDOA, ALOX5, DDC, OAT, PIGS, PFKP, and UQCR10, are related to metabolic pathway. More comparison between CTCs datasets and suspension cells developed from different types of breast cancer cell lines would many more authentic gene list related to metabolic reprogramming as well as CTCs biomarker.

## DISCUSSION

In general, a short-term suspension culture is used for cultivation of CSCs or for mammosphere formation in breast cancer cells. For the enrichment of CSCs, stem cell culture medium is used, which is consisted of serum free DMEM/F12 (1:1) supplemented with basic fibroblast growth factor, epidermal growth factor, insulin, transferrin, and selenium. However, we thought that long-term cultured suspension cells is more similar with circulating tumor cells compared to short-term cultured stem cells. Thus, our suspension cells were maintained with RPMI supplemented with 10% FBS in ultra-low attachment plates without the enrichment of stem cells more than 40 passage and then used to characterize them. In near future, we will characterize suspension cells cultured more than a year.

Although it is best strategy to make suspension cells using primary human tissues, continuous breast cancer cell lines were used to develop suspension cells because of difficulty of making a cell line and availability of tumor tissues. On the other hand, changes in culture conditions dramatically influence cell morphology, cell-matrix interactions, and cell polarity [32, 33] as well as alter gene expression [34]. Thus, more than 40 passages suspension cells were used to avoid the observation of transient phenotypic and genotypic changes in suspension cells. Suspension cells showed slow proliferation rate compared to adherent cells because of increase in G1 population without the significant difference of apoptotic death rate, indicating that more than 40 passages suspension cells were genetically stabilized. On the other hand, because suspension cells showed higher metastatic potential in orthotopic xenograft model, although the proliferation rate of suspension cells was slower than adherent cells, it is likely that suspension cells overcome EMT programming-limited cell proliferation for colonization at the distant metastatic site [35]. These data prove that our suspension cell model appropriate to investigate CTCs and CSCs.

High Vimentin expression was more frequent in matched lymph node and distant metastases compared to that in primary tumors [36] and it was a nonreversible change. Given that Vimentin expression increased in suspension cells, suspension cells are likely to acquire high metastatic potential. Unexpectedly, E-cadherin expression increased in suspension cells, but it is known that E-cadherin expression is reversible depending on the environment [36], and EMT marker Twist represses the expression of E-cadherin [37]. Thus, no Twist expression in suspension cells would be a cause of the increase in E-cadherin expression. On the other hand, Zheng X. *et al.* proved that Snail- or Twist-induced EMT is not rate-limiting for invasion and metastasis by generating mouse models of PDAC with deletion of Snail or Twist [23], although EMT contributes to early-stage dissemination of cancer cells and is pivotal for invasion and metastasis. Therefore, down-regulation of Twist in suspension cells could not weaken metastatic ability.

The two principal energy sources supporting the survival of mammalian cells are glucose and glutamine. Glutamine is converted into glutamate in the cytosol, and the resulting glutamate cannot exit the cell because of its negative charge and, as it accumulates, it promotes tricarboxylic acid (TCA) cycle anaplerosis. Thus, cells are not able to proliferate in cell culture if glutamine is absent [38], and glutamine starvation induces cancer cell death [39]. As suspension cells showed an increase in glutamate, and GLS inhibitor treatment inhibited the proliferation of suspension cells, glutamine-derived glutamate was an important nutrient in suspension cells. Thus, GLS would be a crucial therapeutic target to induce the death of CTCs.

Leucine level was also increased in suspension cells. Catabolism of amino acids falls into three categories: glucogenic, ketogenic, or glucogenic and ketogenic. Glucogenic amino acids, including glutamate, give rise to a net production of pyruvate or TCA cycle intermediates. Two essential amino acids, lysine and leucine, are the only amino acids that are solely ketogenic, giving rise only to acetyl-CoA or acetoacetyl-CoA, which are consumed to generate ATP through TCA cycle. On the other hand, mammalian target of rapamycin complex 1 (mTORC1) protein kinase coordinates cell anabolism and catabolism based on the availability of key nutrients like amino acids. Leucine has the ability to promote activation of mTORC1 [40]. Collectively, leucine increase in suspension cells may be involved in the coordination of mTORC1 activity.

PI (18:0/20:4) has been previously considered as a potential breast cancer biomarker since it significantly decreased in the breast cancer group [41]. Since PI3K is mutated, amplified, and overexpressed in breast cancer [42], highly activated PI3K/serine-threonine kinase (AKT) pathway may undoubtedly lead to a decrease in PIs in cancer cells. However, in this study, PI (18:0/20:4) increased in suspension cells. Thus, it would be important to determine whether suspension cells show decrease in the PI3K/AKT pathway.

Several studies have reported lipid profile changes in cancer cells. The levels of PS (18:0/20:4), PI (18:0/20:4), and PC (18:0/20:4) were markedly higher in metastatic MDA-MB-231 cells than in non-metastatic MCF-7 cells [10]. PI and PE levels were reduced in colitis-associated tumors, and the relative quantities of several PC species decreased in colitis-associated tumor bearing mice fed either diet [43]. Cisplatin- and doxorubicin- resistant MCF-7 cells showed an increase in levels of sphingomyelin, PS, and PA, and a decrease in levels of PE and PC [44]. As it is difficult to elucidate the role of each phospholipid species due to the lack of analysis tools, these changes may be considered as a biomarker to define cellular characteristics. Nonetheless, it is clear that these phospholipid changes contribute to the modulation of membrane fluidity or to the membrane receptor environment, leading to altered cellular adaptation to environmental changes by differently responding to ligand or extracellular matrices. Further studies are required to elucidate which enzyme is responsible for the change of a specific phospholipid species, and which phospholipid species can be an authentic biomarker.

PC levels increases in tumorigenic and highly metastatic breast cancer cells such as MDA-MB-435 and MDA-MB-231 compared to human mammary epithelial cells (HMEC) and their immortalized non-tumoral cell variants [45, 46]. In this study we showed that PC levels increased in the suspension cells compared to adherent cells. Since the suspension cells acquired the more increased metastatic potential, PC increase can be an authentic biomarker for metastatic breast cancer and the regulation of PC level could be a therapeutic target. On the other hand, phosphatidyl lipids have the attached acyl chain of different length and saturation degree. PC containing long chain and PUFA increased in the suspension cells, but PS containing the same fatty acid decreased. Thus, in addition to the increase of PC level, PC species containing long chain fatty acid and PUFA could be more authentic and specific biomarker for highly metastatic cancer.

In summary, we generated suspension cells to mimic CTCs and characterized them by transcriptome, metabolic, and lipidomic analyses. Suspension cells shifted their energy source into glutamate, reduced cholesterol synthesis, and acquired the increased metastatic potential. Since the survival of suspension cells is resistant to the inhibition of cholesterol synthesis and sensitive to the inhibition of GLS, blocking glutamine metabolism would be a promising approach to eradicate CTCs in the blood of patients. The study of metabolic changes in adherent and suspension cells would reveal new principles underlying the mechanism by which the metabolism is orchestrated to support the growth or dormant status of metastatic cells such as CTCs. We believe that this suspension cell model would provide an opportunity to identify a novel therapeutic target for the elimination of CTCs.

## MATERIALS AND METHODS

### Chemicals and reagents

Butylated hydroxytoluene (BHT), myristic-d_27_ acid, methoxylamine hydrochloride, and pyridine were purchased from Sigma-Aldrich (St. Louis, MO, USA). *N*-*O*-bis-(trimethylsilyl)-trifluoroacetamide (BSTFA) containing 1% trimethylchlorosilane (TMCS) was purchased from Alfa Aesar (Ward Hill, MA, USA). High-performance liquid chromatography (HPLC)-grade methanol, chloroform, and water were purchased from Fisher Scientific (Pittsburgh, PA, USA). HPLC-grade hexane was purchased from Honeywell Burdick & Jackson (Muskegon, MI, USA). Bis-2-(5-phenylacetamido-1,3,4-thiadiazol-2-yl)ethyl sulfide (BPTES) and simvastatin were purchased from Sigma-Aldrich. Rabbit anti-glutaminase antibody was purchased from Abcam (San Francisco, CA, USA).

### Generation of suspension cells

MDA-MB-468 human breast cancer cells were purchased from American Type Culture Collection (Manassas, VA, USA). MDA-MB-468 suspension cells were generated and expanded in RPMI 1640 medium (Hyclone, Logan, UT, USA) supplemented with 10% FBS (Equitech-Bio, Kerrville, TX, USA). To synchronize cells in mitosis, cells were treated with 0.1 μg/mL nocodazole for 16 h and then harvested by mechanical shake-off. The cells washed with PBS were plated in ultra-low attachment plates (Corning, Corning, NY, USA). Suspension cells were seeded at density of 2 x 10^5^ cells/ml in ultra-low attachment plates and then subcultured after 3 days. More than 40 passages cells were used to analyze characteristic of suspension cells.

### Cell cycle analysis

Adherent and suspension cells were incubated with 500 nM of propidium iodide for 15 min and cell cycle was analyzed using flow cytometry.

### Real-time polymerase chain reaction (RT-PCR) analysis

The primers used for RT-PCR amplification were: E-cadherin forward (5′-TTCCTCCCAATACAT CTCCC-3′), E-cadherin reverse (5′-TTGATTTTGTAGTC ACCCACC-3′), Twist forward (5′-GTCCGCAGTCTT ACGAGGAG-3′), Twist reverse (5′-GCTTGAGGGTC TGAATCTTGCT-3′), Vimentin forward (5′-CTCTTCC AAACTTTTCCTCCC-3′), Vimentin reverse (5′- AGTTTC GTTGATAACCTGTCC-3′), Snail forward (5′-CAGACC CACTCAGATGTCAA-3′), Snail reverse (5′-CATAGTTA GTCACACCTCGT-3′), Slug forward (5′- GGTCAAG AAGCATTTCAAC-3′), and Slug reverse (5′-GGTAAT GTGTGGGTCCGA-3′) Alu forward (5′-ACGCCTGT AATCCCAGCACTT-3′), Alu reverse (5′-TCGCCCAGGC TGGAGTGCA-3′), mGAPDH forward (5′-GCACAGT CAAGGCCGAGAAT-3′), mGAPDH reverse (5′-GCC TTCTCCATGGTGGTGAA-3′).

### Animal experiments

For stable transfection of firefly luciferase, viruses were produced by transfecting plasmids in HEK 293T cells and the 2nd generation of the lentiviral system was used. After collecting the virus, MDA-MB-468 adherent and suspension cells were infected and selected by puromycin (0.5 μg/ml). For inducing mammary tumor formation, 5 × 10^5^ of luciferase-expressing adherent or suspension cells were suspended in 50 μl of 1:1 mix of phosphate buffered saline (PBS) and matrigel (BD Biosciences, Bedford, MA, USA) injected into the right inguinal mammary fat pad of 6-week-old female athymic nu/nu mice (Saeronbio Inc., Kyunggi-do, Republic of Korea). For *in vivo* bioluminescence imaging, mice were given an intraperitoneal (i.p.) injection with 100 μl of D-luciferin dissolved in PBS (30 mg/ml, Promega, Madison, WI, USA). Mice were anesthetized with isoflurane (2% in 1L/min oxygen), and bioluminescence images were acquired 13 minutes after D-luciferin injection using the IVIS Lumina XRMS (Caliper Life Sciences, Hopkinton, MA, USA). After 12 weeks, mice were euthanized and metastases in lung or liver were qualified by histological staining with anti-vimentin antibody (Cell signaling, #5741). For lung colonization experiments, 1 × 10^6^ adherent and suspension cells were injected into the tail vein of 6-week-old female NOD-scid-gamma mice (NSG, The Jackson Laboratory, Bar Harbor, ME, USA). After 10 weeks, mice were euthanized and metastases in lung were qualified by histological staining with anti-vimentin antibody. Plans and protocols for animal experiments were approved by the Institutional Animal Care and Use Committee of Sookmyung Women’s University, Seoul, Republic of Korea (SMWU-IACUC-1608-018).

### Circulating tumor cell analysis

To measure tumor cells in blood, blood was drawn by performing cardiac puncture following genomic DNA isolation using a commercial kit (Norgen Biotek, Thorold, ON, Canada). Real time PCR reactions were performed on the genomic DNA as the template with primers against human Alu, which amplifies DNA segment from circulating human breast cancer cells, and mouse glyceraldehyde 3-phosphate dehydrogenase (GAPDH), which amplifies DNA segment from mouse blood cells. Level of CTCs were determined by amount of human Alu normalized with mouse GAPDH.

### RNA-seq analysis

MDA-MB-468 adherent and suspension cells, which were passaged more than 40 times, were used for the analysis. Total RNA was extracted from MDA-MB-468 adherent and suspension cells, and sequencing of the prepared library was conducted by LAS Inc., Seoul, Republic of Korea.

### Non-targeted gas chromatography-mass spectrometry (GC-MS) for metabolite analysis

Metabolites were extracted from adherent and suspension cells according to a previous report [10]. To perform derivatization of the extracted sample, 30 µL of 20,000 µg/mL methoxyamine hydrochloride in pyridine, 50 µL of BSTFA containing 1% TMCS, and 10 µL of myristic-d_27_ acid were added to dried samples. The samples were then incubated for 60 min at 65°C. Finally, each extracted sample was used for GC-MS analysis.

### Nanoelectro-spray ionization tandem mass spectrometry (NanoESI-MS) for lipid analysis

Lipid extracts were analyzed in positive and negative ion modes with nanoESI-MS using a linear ion trap mass spectrometer (LTQ-XL; Thermo Fisher Scientific, San Jose, CA, USA) equipped with an automated nanoinfusion/nanospray source (TriVersa NanoMate System; Advion Biosciences, Ithaca, NY, USA). Lipid species were identified based on the tandem mass spectrometry (MS/MS) spectra of an authentic reference and an in-house MS/MS library. Lipid Maps (http://www.lipidmaps.org) and LipidBlast databases were used to match the spectra. The identification of ceramide species was based on authentic reference MS/MS spectra by Han [47].

### Data processing and statistical analysis

The raw data files were processed with Expressionist MSX software (version 2013.0.39; Genedata, Basel, Switzerland) for the relative quantification of metabolomes and lipidomes. The data were normalized by dividing the peak intensity of the internal standard by the total protein content. Fold changes and Student’s *t*-tests (at a threshold of *p* < 0.05) were assessed using MetaboAnalyst (http://www.metaboanalyst.ca). For multivariate statistical analysis, principal component analysis (PCA) was performed using SIMCA-P+ software using mean-centered and unit variance-scaled data.

## SUPPLEMENTARY MATERIALS FIGURES AND TABLES







## Abbreviations

CTCs: Circulating tumor cells
EMT: Epithelial mesenchymal transition
CSCs: Cancer stem cells
ERα: Estrogen receptor α
PR: Progesterone receptor
HER2: Human epidermal growth factor receptor 2
EpCAM: Epithelial cellular adhesion molecule
GLS: Glutaminase
PC: Phosphatidylcholine
PE: Phosphatidylethanolamine
CER: Ceramide
PG: Phosphatidylglycerol
PS: Phosphatidylserine
PI: Phosphatidylinositol
PUFA: poly unsaturated fatty acids
KEGG: Kyoto Encyclopedia of Genes and Genomes
SCD1: Stearoyl-CoA desaturase-1
SCD5: Stearoyl-CoA desaturase -5
ELOVL 1/3/7: Elongation of very long chain fatty acids
PLA2G6: Phospholipase A2 Group VI
TCA: Tricarboxylic acid
mTORC1: Mammalian target of rapamycin complex 1
AKT: Activated PI3K/serine-threonine kinase
HMEC: Human mammary epithelial cells
