# Supplementary material for: Development of suspension cell culture model to mimic circulating tumor cells
Source: Oncotarget. 2017 Dec 7;9(1):622–40. doi: 10.18632/oncotarget.23079 (PMC5787494; doi:10.18632/oncotarget.23079)
Supplement: Supplementary file 1 [file oncotarget-09-622-s001.pdf]

Development of suspension cell culture model to mimic circulating tumor cells

SUPPLEMENTARY MATERIALS

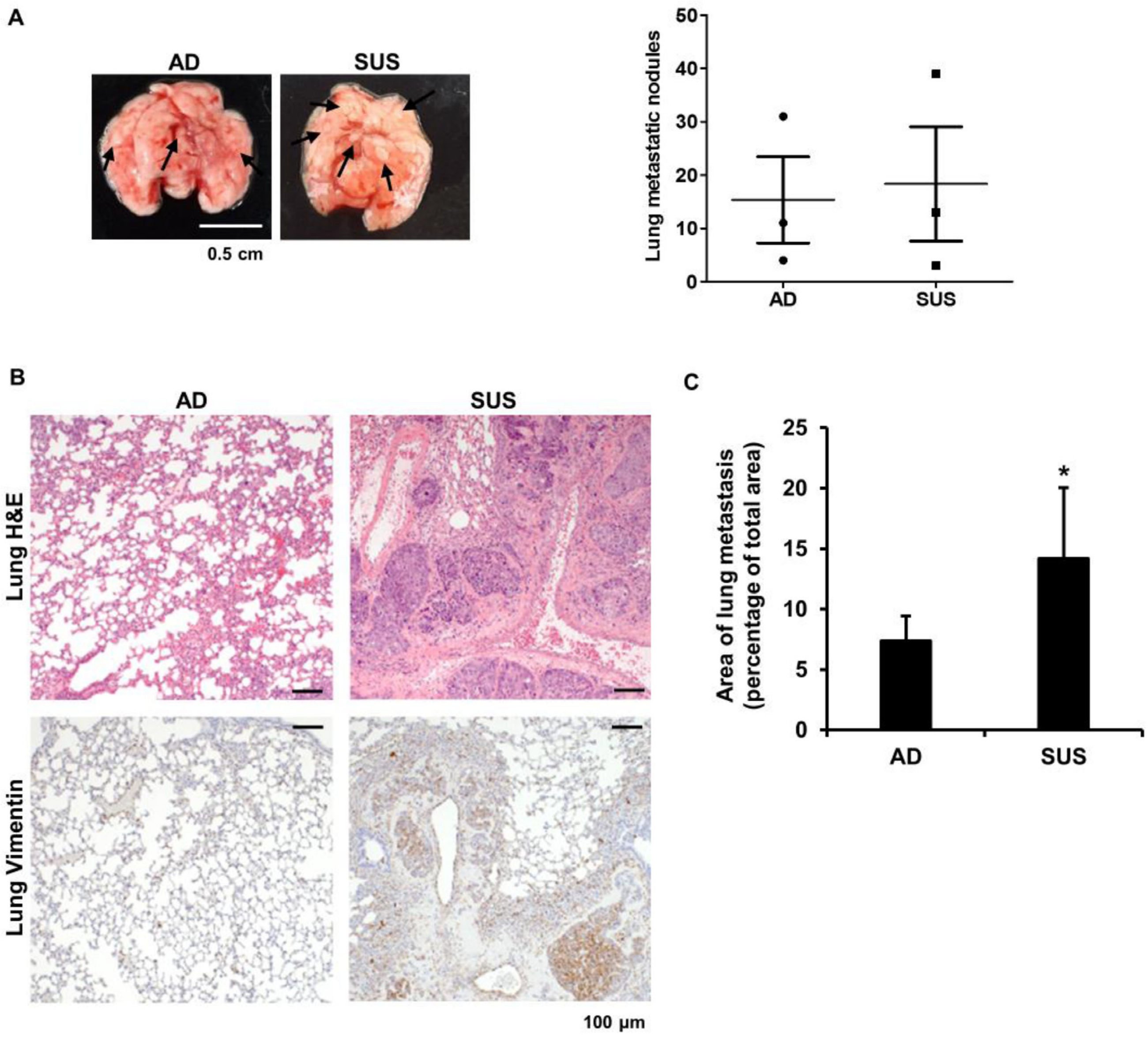

Supplementary Figure 1: Suspension cells show higher metastatic area in lung colonization. (**A**) MDA-MB-468 adherent or suspension cells were directly injected into the lateral tail vein of female NOD-scid-gamma (NSG) mice. Representative gross morphology of lungs at 10 weeks after tail-vein injection of adherent or suspension cells are shown. Arrows indicate metastatic nodules. Metastatic tumor nodules in the lung were counted and data are shown as means ± SEM, $n = 6$. (**B**) Representative images of lung sections staining with vimentin in two groups of animals are shown (Scale bar = 100 µm). (**C**) The percentage of micro-metastasis area in two groups are shown. $^{*}p < 0.05$; one-tailed Student's t-test.

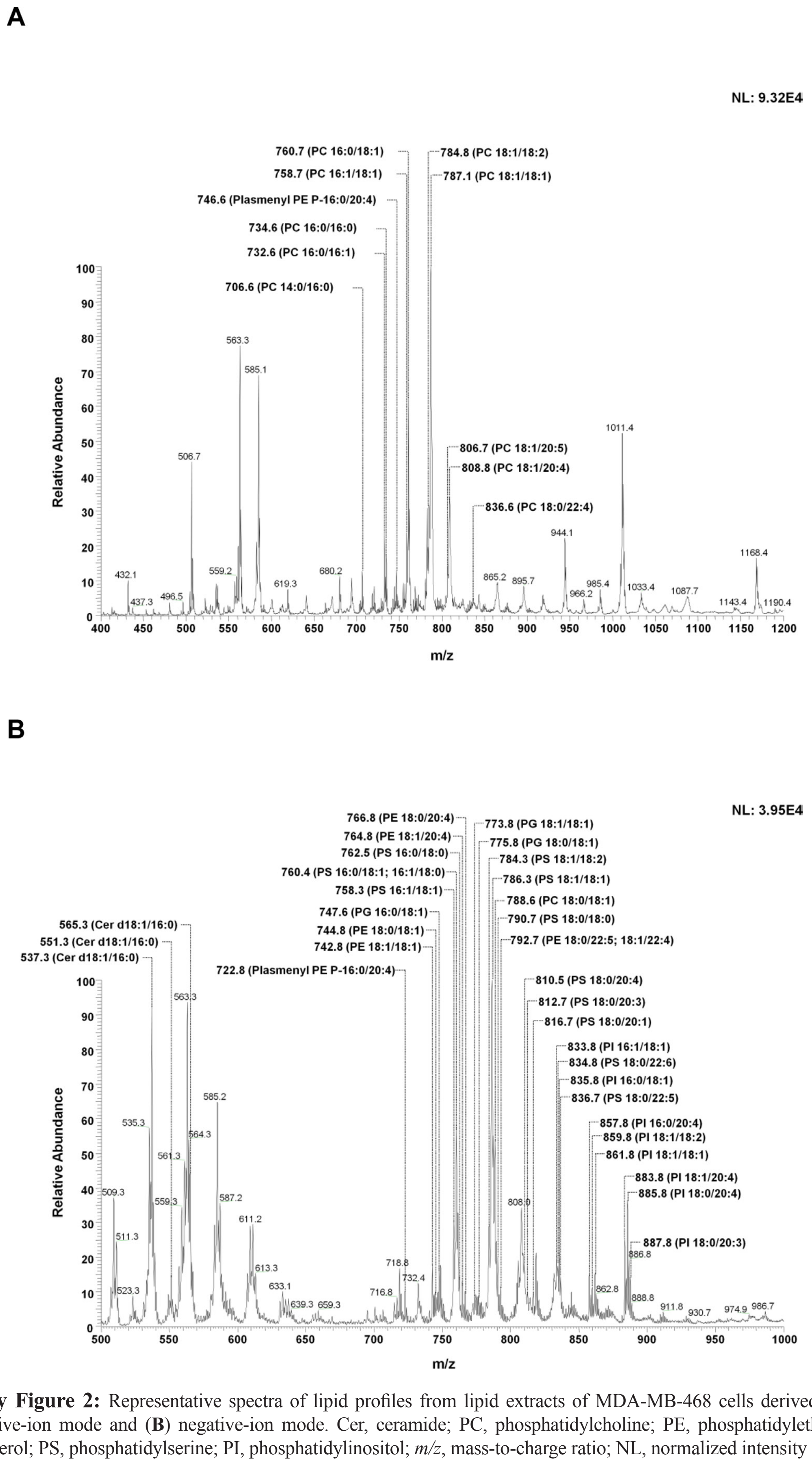

Supplementary Figure 2: Representative spectra of lipid profiles from lipid extracts of MDA-MB-468 cells derived from nanoESI-MS in (**A**) positive-ion mode and (**B**) negative-ion mode. Cer, ceramide; PC, phosphatidylcholine; PE, phosphatidylethanolamine; PG, phosphatidylglycerol; PS, phosphatidylserine; PI, phosphatidylinositol; *m/z*, mass-to-charge ratio; NL, normalized intensity level.

Supplementary Table 1: Lipid species identified from lipid extracts of adherent and suspension MDA-MB-468 cells using nanoESI-tandem MS analyses. See Supplementary_Table_1

Supplementary Table 2: Commonly up- and down-regulated genes between suspension MDA-MB-468 cells and patient CTCs. See Supplementary_Table_2
