# Supplementary material for: Development of suspension cell culture model to mimic circulating tumor cells
Source: Oncotarget. 2017 Dec 7;9(1):622–40. doi: 10.18632/oncotarget.23079 (PMC5787494; doi:10.18632/oncotarget.23079)
Supplement: Supplementary file 2 [file oncotarget-09-622-s002.docx]

**Supplementary Table 1: Lipid species identified from lipid extracts of adherent and suspension MDA-MB-468 cells using nanoESI-tandem MS analyses**

| Lipid molecular species | Ion species | | *m/z* | | MS/MS fragment ion (*m/z*) | |
| --- | --- | --- | --- | --- | --- | --- |
| Positive ion mode |  | |  | |  | |
| Phosphatidylcholine (PC) | | |  | |  | |
| PC (14:0/16:0) | [M + H]^+^ | | 706.6 | | 450 [lyso-PC(14:0) – H_2_O + H]^+^; 468 [lyso-PC(14:0) + H]^+^; 478 [lyso-PC(16:0) – H_2_O + H]^+^; 496 [lyso-PC(16:0) + H]^+^; 523 [M – C_5_H_14_NO_4_P + H]^+^; 647 [M – C_3_H_9_N + H]^+^; 688 [M – H_2_O + H]^+^ | |
| PC (16:0/16:1) | [M + H]^+^ | | 732.6 | | 476 [lyso-PC(16:1) – H_2_O + H]^+^; 478 [lyso-PC(16:0) – H_2_O + H]^+^; 494 [lyso-PC(16:1) + H]^+^; 496 [lyso-PC(16:0) + H]^+^; 549 [M – C_5_H_14_NO_4_P + H]^+^; 673 [M – C_3_H_9_N + H]^+^; 714 [M – H_2_O + H]^+^ | |
| PC (16:0/16:0) | [M + H]^+^ | | 734.6 | | 478 [lyso-PC(16:0) – H_2_O + H]^+^; 496 [lyso-PC(16:0) + H]^+^; 551 [M – C_5_H_14_NO_4_P + H]^+^; 675 [M – C_3_H_9_N + H]^+^; 716 [M – H_2_O + H]^+^ | |
| PC (16:1/18:1) | [M + H]^+^ | | 758.7 | | 476 [lyso-PC(16:1) – H_2_O + H]^+^; 494 [lyso-PC(16:1) + H]^+^; 504 [lyso-PC(18:1) – H_2_O + H]^+^; 522 [lyso-PC(18:1) + H]^+^; 575 [M – C_5_H_14_NO_4_P + H]^+^; 699 [M – C_3_H_9_N + H]^+^; 740 [M – H_2_O + H]^+^ | |
| PC (16:0/18:1) | [M + H]^+^ | | 760.7 | | 478 [lyso-PC(16:0) – H_2_O + H]^+^; 496 [lyso-PC(16:0) + H]^+^; 504 [lyso-PC(18:1) – H_2_O + H]^+^; 522 [lyso-PC(18:1) + H]^+^; 577 [M – C_5_H_14_NO_4_P + H]^+^; 701 [M – C_3_H_9_N + H]^+^; 742 [M – H_2_O + H]^+^ | |
| PC (18:1/18:2) | [M + H]^+^ | | 784.8 | | 502 [lyso-PC(18:2) – H_2_O + H]^+^; 504 [lyso-PC(18:1) – H_2_O + H]^+^; 520 [lyso-PC(18:2) + H]^+^; 522 [lyso-PC(18:1) + H]^+^; 601 [M – C_5_H_14_NO_4_P + H]^+^; 725 [M – C_3_H_9_N + H]^+^; 766 [M – H_2_O + H]^+^ | |
| PC (18:1/18:1) | [M + H]^+^ | | 787.1 | | 504 [lyso-PC(18:1) – H_2_O + H]^+^; 522 [lyso-PC(18:1) + H]^+^; 603 [M – C_5_H_14_NO_4_P + H]^+^; 727 [M – C_3_H_9_N + H]^+^; 768 [M – H_2_O + H]^+^ | |
| PC (18:1/20:5) | [M + H]^+^ | | 806.7 | | 504 [lyso-PC(18:1) – H_2_O + H]^+^; 524 [lyso-PC(20:5) – H_2_O + H]^+^; 623 [M – C_5_H_14_NO_4_P + H]^+^; 747 [M – C_3_H_9_N + H]^+^; 788 [M – H_2_O + H]^+^ | |
| PC (18:1/20:4) | [M + H]^+^ | | 808.8 | | 504 [lyso-PC(18:1) – H_2_O + H]^+^; 526 [lyso-PC(20:4) – H_2_O + H]^+^; 522 [lyso-PC(18:1) + H]^+^; 625 [M – C_5_H_14_NO_4_P + H]^+^; 749 [M – C_3_H_9_N + H]^+^; 790 [M – H_2_O + H]^+^ | |
| PC (18:0/22:5) | [M + H]^+^ | | 836.6 | | 552 [lyso-PC(22:5) – H_2_O + H]^+^ ; 653 [M – C_5_H_14_NO_4_P + H]^+^; 777 [M – C_3_H_9_N + H]^+^; 818 [M – H_2_O + H]^+^ | |
| Phosphatidylethanolamine (PE) | | |  | |  | |
| PE (P-16:0/20:4) | [M + Na]^+^ | | 746.6 | | 386 [P-16:0 + C_2_H_8_NO_3_P + Na]^+^; 605 [M – C_2_H_8_NO_4_P + Na]^+^; 623 [M – C_2_H_8_NO_4_P + H_2_O + Na]^+^; 703 [M – C_2_H_5_N + Na]^+^; 728 [M – H_2_O + Na]^+^ | |
| Negative ion mode |  | |  | |  | |
| Ceramide (Cer) | | |  | |  | |
| Cer (d18:1/16:0) | [M – H]^–^ | | 537.3 | | 519 [M – H_2_O – H]^–^; 507 [M – H – 30]^–^; 505 [M – H – 32]^–^; 489 [M – H – 48]^–^; 297 [M – H – 240] ^–^; 281 [M – H – 256]^–^; 255 [M – H – 282]^–^ | |
| Cer (d18:1/17:0) | [M – H]^–^ | | 551.3 | | 238 [M – H – 299]^–^; 532 [M – H_2_O – H]^–^; 520 [M – H – 30]^–^; 518 [M – H – 32]^–^; 502 [M – H – 48]^–^; 295 [M – H – 256]^–^; 269 [M – H – 281]^–^; 268 [M – H – 282]^–^ | |
| Cer (d18:1/18:0) | [M – H]^–^ | | 565.3 | | 547 [M – H_2_O – H]^–^; 535 [M – H – 30]^–^; 532 [M – H – 32]^–^; 516 [M – H – 48]^–^; 324 [M – H – 240]^–^ | |
| Phosphatidylethanolamine (PE) | | |  | |  | |
| PE (P-16:0/20:4) | [M – H]^–^ | | 722.8 | | 303 [C20:4 – H]^–^; 418 [lyso-PE(P-16:0) – H_2_O – H]^–^; 436 [lyso-PE(P-16:0) – H]^–^ | |
| PE (18:1/18:1) | [M – H]^–^ | | 742.8 | | 281 [C18:1 – H]^–^; 460 [lyso-PE(18:1) – H_2_O – H]^–^; 478 [lyso-PE(18:1) – H]^–^ | |
| PE (18:0/18:1) | [M – H]^–^ | | 744.8 | | 281 [C18:1 – H]^–^; 283 [C18:0 – H]^–^; 460 [lyso-PE(18:1) – H_2_O – H]^–^; 462 [lyso-PE(18:0) – H_2_O – H]^–^; 478 [lyso-PE(18:1) – H]^–^; 480 [lyso-PE(18:0) – H]^–^ | |
| PE (18:1/20:4) | [M – H]^–^ | | 764.8 | | 281 [C18:1 – H]^–^; 303 [C20:4 – H]^–^; 460 [lyso-PE(18:1) – H_2_O – H]^–^; 478 [lyso-PE(18:1) – H]^–^; 482 [lyso-PE(20:4) – H_2_O – H]^–^; 500 [lyso-PE(20:4) – H]^–^ | |
| PE (18:0/20:4) | [M – H]^–^ | | 766.8 | | 283 [C18:0 – H]^–^; 303 [C20:4 – H]^–^; 462 [lyso-PE(18:0) – H_2_O – H]^–^; 480 [lyso-PE(18:0) – H]^–^; 482 [lyso-PE(20:4) – H_2_O – H]^–^; 500 [lyso-PE(20:4) – H]^–^ | |
| PE (18:0/22:5) | [M – H]^–^ | | 792.7 | | 283 [C18:0 – H]^–^; 329 [C22:5 – H]^–^; 462 [lyso-PE(18:0) – H_2_O – H]^–^; 480 [lyso-PE(18:0) – H]^–^; 508 [lyso-PE(22:5) – H_2_O – H]^–^; 526 [lyso-PE(22:5) – H]^–^ | |
| PE (18:1/22:4) | [M – H]^–^ | | 792.7 | | 281 [C18:1 – H]^–^; 331 [C22:4 – H]^–^; 460 [lyso-PE(18:1) – H_2_O – H]^–^; 482 [lyso-PE(18:1) – H]^–^; 528 [lyso-PE(22:4) – H]^–^ | |
| Phosphatidylglycerol (PG) | | |  | |  | |
| PG (16:0/18:1) | [M – H]^–^ | | 747.6 | | 255 [C16:0 – H]^–^; 281 [C18:1 – H]^–^; 391 [lyso-PG(16:0) – C_3_H_6_O_2_ – H]^–^; 417 [lyso-PG(18:1) – C_3_H_6_O_2_ – H]^–^; 465 [lyso-PG(16:0) – H_2_O – H]^–^; 483 [lyso-PG(16:0) – H]^–^; 491 [lyso-PG(18:1) – H_2_O – H]^–^; 509 [lyso-PG(18:1) – H]^–^ | |
| PG (18:1/18:1) | [M – H]^–^ | | 773.8 | | 281 [C18:1 – H]^–^; 417 [lyso-PG(18:1) – C_3_H_6_O_2_ – H]^–^; 491 [lyso-PG(18:1) – H_2_O – H]^–^; 509 [lyso-PG(18:1) – H]^–^ | |
| PG (18:0/18:1) | [M – H]^–^ | | 775.8 | | 281 [C18:1 – H]^–^; 283 [C18:0 – H]^–^; 417 [lyso-PG(18:1) – C_3_H_6_O_2_ – H]^–^; 419 [lyso-PG(18:0) – C_3_H_6_O_2_ – H]^–^; 491 [lyso-PG(18:1) – H_2_O – H]^–^; 493 [lyso-PG(18:0) – H_2_O – H]^–^; 509 [lyso-PG(18:1) – H]^–^; 511 [lyso-PG(18:0) – H]^–^ | |
| Phosphatidylserine (PS) | | |  | |  | |
| PS (16:1/18:1) | [M – H]^–^ | | 758.3 | | 253 [C16:1 – H]^–^; 281 [C18:1 – H]^–^; 417 [M – C_3_H_5_NO_2_ – H – C16:1 – H_2_O]^–^; 671 [M – C_3_H_5_NO_2_ – H]^–^ | |
| PS (16:0/18:1) | [M – H]^–^ | | 760.4 | | 255 [C16:0 – H]^–^; 281 [C18:1 – H]^–^; 391 [M – C_3_H_5_NO_2_ – H – C18:1 – H_2_O]^–^; 409 [M – C_3_H_5_NO_2_ – H – C18:1]^–^; 417 [M – C_3_H_5_NO_2_ – H – C16:0 – H_2_O]^–^; 435 [M – C_3_H_5_NO_2_ – H – C16:0]^–^; 673 [M – C_3_H_5_NO_2_ – H]^–^ | |
| PS (16:1/18:0) | [M – H]^–^ | | 760.4 | | 253 [C16:1 – H]^–^; 283 [C18:0 – H]^–^; 389 [M – C_3_H_5_NO_2_ – H – C18:0 – H_2_O]^–^; 419 [M – C_3_H_5_NO_2_ – H – C16:1 – H_2_O]^–^; 437 [M – C_3_H_5_NO_2_ – H – C16:1]^–^; 673 [M – C_3_H_5_NO_2_ – H]^–^ | |
| PS (16:0/18:0) | [M – H]^–^ | | 762.5 | | 255 [C16:0 – H]^–^; 283 [C18:0 – H]^–^; 391 [M – C_3_H_5_NO_2_ – H – C18:0 – H_2_O]^–^; 409 [M – C_3_H_5_NO_2_ – H – C18:0]^–^; 419 [M – C_3_H_5_NO_2_ – H – C16:0 – H_2_O]^–^; 437 [M – C_3_H_5_NO_2_ – H – C16:0]^–^; 675 [M – C_3_H_5_NO_2_ – H]^–^ | |
| PS (18:1/18:2) | [M – H]^–^ | | 784.3 | | 279 [C18:2 – H]^–^; 281 [C18:1 – H]^–^; 415 [M – C_3_H_5_NO_2_ – H – C18:1 – H_2_O]^–^; 417 [M – C_3_H_5_NO_2_ – H – C18:2 – H_2_O]^–^; 433 [M – C_3_H_5_NO_2_ – H – C18:1]^–^; 435 [M – C_3_H_5_NO_2_ – H – C18:2]^–^; 697 [M – C_3_H_5_NO_2_ – H]^–^ | |
| PS (18:1/18:1) | [M – H]^–^ | | 786.3 | | 281 [C18:1 – H]^–^; 417 [M – C_3_H_5_NO_2_ – H – C18:1 – H_2_O]^–^; 435 [M – C_3_H_5_NO_2_ – H – C18:1]^–^; 699 [M – C_3_H_5_NO_2_ – H]^–^ | |
| PS (18:0/18:1) | [M – H]^–^ | | 788.6 | | 281 [C18:1 – H]^–^; 283 [C18:0 – H]^–^; 417 [M – C_3_H_5_NO_2_ – H – C18:0 – H_2_O]^–^; 419 [M – C_3_H_5_NO_2_ – H – C18:1 – H_2_O]^–^; 435 [M – C_3_H_5_NO_2_ – H – C18:0]^–^; 437 [M – C_3_H_5_NO_2_ – H – C18:1]^–^; 701 [M – C_3_H_5_NO_2_ – H]^–^ | |
| PS (18:0/18:0) | [M – H]^–^ | | 790.7 | | 283 [C18:0 – H]^–^; 419 [M – C_3_H_5_NO_2_ – H – C18:0 – H_2_O]^–^; 437 [M – C_3_H_5_NO_2_ – H – C18:0]^–^; 703 [M – C_3_H_5_NO_2_ – H]^–^ | |
| PS (18:0/20:4) | [M – H]^–^ | | 810.5 | | 283 [C18:0 – H]^–^; 303 [C20:4 – H]^–^; 419 [M – C_3_H_5_NO_2_ – H – C20:4 – H_2_O]^–^; 437 [M – C_3_H_5_NO_2_ – H – C20:4]^–^; 439 [M – C_3_H_5_NO_2_ – H – C18:0 – H_2_O]^–^; 457 [M – C_3_H_5_NO_2_ – H – C18:0]^–^; 723 [M – C_3_H_5_NO_2_ – H]^–^ | |
| PS (18:0/20:3) | [M – H]^–^ | | 812.7 | | 283 [C18:0 – H]^–^; 305 [C20:3 – H]^–^; 419 [M – C_3_H_5_NO_2_ – H – C20:3 – H_2_O]^–^; 437 [M – C_3_H_5_NO_2_ – H – C20:3]^–^; 441 [M – C_3_H_5_NO_2_ – H – C18:0 – H_2_O]^–^; 459 [M – C_3_H_5_NO_2_ – H – C18:0]^–^; 725 [M – C_3_H_5_NO_2_ – H]^–^ | |
| PS (18:0/20:1) | [M – H]^–^ | | 816.7 | | 283 [C18:0 – H]^–^; 309 [C20:1 – H]^–^; 419 [M – C_3_H_5_NO_2_ – H – C20:1 – H_2_O]^–^; 437 [M – C_3_H_5_NO_2_ – H – C20:1]^–^; 445 [M – C_3_H_5_NO_2_ – H – C18:0 – H_2_O]^–^; 463 [M – C_3_H_5_NO_2_ – H – C18:0]^–^; 729 [M – C_3_H_5_NO_2_ – H]^–^ | |
| PS (18:0/22:6) | [M – H]^–^ | | 834.8 | | 283 [C18:0 – H]^–^; 327 [C22:6 – H]^–^; 419 [M – C_3_H_5_NO_2_ – H – C22:6 – H_2_O]^–^; 437 [M – C_3_H_5_NO_2_ – H – C22:6]^–^; 463 [M – C_3_H_5_NO_2_ – H – C18:0 – H_2_O]^–^; 747 [M – C_3_H_5_NO_2_ – H]^–^ | |
| PS (18:0/22:5) | [M – H]^–^ | | 836.7 | | 283 [C18:0 – H]^–^; 329 [C22:5 – H]^–^; 419 [M – C_3_H_5_NO_2_ – H – C22:5 – H_2_O]^–^; 437 [M – C_3_H_5_NO_2_ – H – C22:5]^–^; 465 [M – C_3_H_5_NO_2_ – H – C18:0 – H_2_O]^–^; 749 [M – C_3_H_5_NO_2_ – H]^–^ | |
| Phosphatidylinositol (PI) | |  | |  | |  |
| PI (16:1/18:1) | [M – H]^–^ | | 833.8 | | 241 [C_6_H_10_O_8_P]^–^; 253 [C16:1 – H]^–^; 281 [C18:1 – H]^–^; 297 [C_9_H_14_O_9_P]^–^ ; 315 [C_9_H_16_O_10_P]^–^; 389 [lyso-PI(16:1) – C_6_H_12_O_6_ – H]^–^; 417 [lyso-PI(18:1) – C_6_H_12_O_6_ – H]^–^; 551 [lyso-PI(16:1) – H_2_O – H]^–^; 569 [lyso-PI(16:1) – H]^–^ ; 579 [lyso-PI(18:1) – H_2_O – H]^–^ ; 597 [lyso-PI(18:1) – H]^–^ | |
| PI (16:0/18:1) | [M – H]^–^ | | 835.88 | | 241 [C_6_H_10_O_8_P]^–^; 255 [C16:0 – H]^–^; 281 [C18:1 – H]^–^; 297 [C_9_H_14_O_9_P]^–^; 315 [C_9_H_16_O_10_P]^–^; 391 [lyso-PI(16:0) – C_6_H_12_O_6_ – H]^–^; 417 [lyso-PI(18:1) – C_6_H_12_O_6_ – H]^–^; 553 [lyso-PI(16:0) – H_2_O – H]^–^; 571 [lyso-PI(16:0) – H]^–^; 597 [lyso-PI(18:1) – H_2_O – H]^–^ | |
| PI (16:0/20:4) | [M – H]^–^ | | 857.8 | | 241 [C_6_H_10_O_8_P]^–^; 255 [C16:0 – H]^–^; 297 [C_9_H_14_O_9_P]^–^; 303 [C20:4 – H]^–^; 315 [C_9_H_16_O_10_P]^–^; 391 [lyso-PI(16:0) – C_6_H_12_O_6_ – H]^–^; 439 [lyso-PI(20:4) – C_6_H_12_O_6_ – H]^–^; 553 [lyso-PI(16:0) – H_2_O – H]^–^; 571 [lyso-PI(16:0) – H]^–^; 601 [lyso-PI(20:4) – H_2_O – H]^–^; 619 [lyso-PI(20:4) – H]^–^ | |
| PI (18:1/18:2) | [M – H]^–^ | | 859.53 | | 241 [C_6_H_10_O_8_P]^–^; 279 [C18:2 – H]^–^; 281 [C18:1 – H]^–^; 297 [C_9_H_14_O_9_P]^–^; 315 [C_9_H_16_O_10_P]^–^; 415 [lyso-PI(18:2) – C_6_H_12_O_6_ – H]^–^; 417 [lyso-PI(18:1) – C_6_H_12_O_6_ – H]^–^; 577 [lyso-PI(18:2) – H_2_O – H]^–^; 579 [lyso-PI(18:1) – H_2_O – H]^–^; 595 [lyso-PI(18:2) – H]^–^; 597 [lyso-PI(18:1) – H]^–^ | |
| PI (18:1/18:1) | [M – H]^–^ | | 861.8 | | 241 [C_6_H_10_O_8_P]^–^; 281 [C18:1 – H]^–^; 297 [C_9_H_14_O_9_P]^–^; 315 [C_9_H_16_O_10_P]^–^; 417 [lyso-PI(18:1) – C_6_H_12_O_6_ – H]^–^; 579 [lyso-PI(18:1) – H_2_O – H]^–^; 597 [lyso-PI(18:1) – H]^–^ | |
| PI (18:1/20:4) | [M – H]^–^ | | 883.8 | | 281 [C18:1 – H]^–^; 297 [C_9_H_14_O_9_P]^–^; 303 [C20:4 – H]^–^; 315 [C_9_H_16_O_10_P]^–^; 417 [lyso-PI(18:1) – C_6_H_12_O_6_ – H]^–^; 439 [lyso-PI(20:4) – C_6_H_12_O_6_ – H]^–^; 579 [lyso-PI(18:1) – H_2_O – H]^–^; 597 [lyso-PI(18:1) – H]^–^; 601 [lyso-PI(20:4) – H_2_O – H]^–^; 619 [lyso-PI(20:4) – H]^–^ | |
| PI (18:0/20:4) | [M – H]^–^ | | 885.8 | | 283 [C18:0 – H]^–^; 297 [C_9_H_14_O_9_P]^–^; 303 [C20:4 – H]^–^; 315 [C_9_H_16_O_10_P]^–^; 419 [lyso-PI(18:0) – C_6_H_12_O_6_ – H]^–^; 439 [lyso-PI(20:4) – C_6_H_12_O_6_ – H]^–^; 581 [lyso-PI(18:0) – H_2_O – H]^–^; 599 [lyso-PI(18:0) – H]^–^; 601 [lyso-PI(20:4) – H_2_O – H]^–^; 619 [lyso-PI(20:4) – H]^–^ | |
| PI (18:0/20:3) | [M – H]^–^ | | 887.8 | | 283 [C18:0 – H]^–^; 297 [C_9_H_14_O_9_P]^–^; 305 [C20:3 – H]^–^; 315 [C_9_H_16_O_10_P]^–^; 419 [lyso-PI(18:0) – C_6_H_12_O_6_ – H]^–^; 441 [lyso-PI(20:3) – C_6_H_12_O_6_ – H]^–^; 581 [lyso-PI(18:0) – H_2_O – H]^–^; 599 [lyso-PI(18:0) – H]^–^; 603 [lyso-PI(20:3) – H_2_O – H]^–^; 621 [lyso-PI(20:3) – H]^–^ | |
