# Supplementary material for: Development of suspension cell culture model to mimic circulating tumor cells
Source: Oncotarget. 2017 Dec 7;9(1):622–40. doi: 10.18632/oncotarget.23079 (PMC5787494; doi:10.18632/oncotarget.23079)
Supplement: Supplementary file 3 [file oncotarget-09-622-s003.docx]

**Supplementary Table 2: Commonly up- and down-regulated genes between suspension MDA-MB-468 cells and patient CTCs**

| Gene | log2(fold_change) |
| --- | --- |
| FOSB | 7.10286 |
| PDK4 | 3.81755 |
| ABCC6P1 | 2.27386 |
| DKK1 | 2.27339 |
| SLC43A1 | 2.15619 |
| LOC100272228 | 1.72803 |
| DUSP10 | 1.36289 |
| FGL1 | 1.35634 |
| ZIC2 | 1.22323 |
| ZNF292 | 1.21881 |
| TUBE1 | 1.20618 |
| ZNF680 | 1.18449 |
| RHOBTB3 | 1.1791 |
| INA | 1.04233 |
| ZNF202 | 1.03235 |
| JMY | 1.02674 |
| DDC | 1.01952 |
| ITGBL1 | -10.7638 |
| ISLR | -4.87718 |
| OLFML2A | -3.72362 |
| SLC39A2 | -3.68729 |
| ID3 | -2.91499 |
| SAA1 | -2.90581 |
| CCDC80 | -2.57706 |
| LHFP | -2.48144 |
| FAP | -2.3084 |
| PSMB8 | -1.98603 |
| UBE2L6 | -1.9324 |
| FN1 | -1.92941 |
| ATOX1 | -1.89864 |
| GM2A | -1.7243 |
| LGALS3BP | -1.71656 |
| IGFBP5 | -1.57628 |
| PSME2 | -1.52784 |
| MGST2 | -1.5233 |
| RGS10 | -1.5179 |
| TMSB4X | -1.47903 |
| MYL6 | -1.47792 |

| ARPC1B | -1.4749 |
| --- | --- |
| FLOT2 | -1.38877 |
| COL4A2 | -1.38856 |
| C1R | -1.37408 |
| MAPK3 | -1.36487 |
| NDUFS5 | -1.34665 |
| CSTB | -1.28074 |
| OAZ1 | -1.24565 |
| UQCR10 | -1.24094 |
| DYNLL1 | -1.23789 |
| ALOX5 | -1.2341 |
| HLA-C | -1.2216 |
| LAGE3 | -1.22024 |
| ATP5G3 | -1.21403 |
| OAT | -1.20197 |
| NDUFB1 | -1.18578 |
| FAM96A | -1.1815 |
| MARCKS | -1.16587 |
| ALDOA | -1.16368 |
| HLA-A | -1.14819 |
| PLIN3 | -1.14385 |
| PTRF | -1.14228 |
| PIGS | -1.11575 |
| LOC550643 | -1.09114 |
| TAGLN2 | -1.09038 |
| MRPS7 | -1.08446 |
| NTAN1 | -1.0805 |
| PSMD2 | -1.07286 |
| PFKP | -1.06635 |
| PPP1CA | -1.05558 |
| QSOX1 | -1.02347 |
| DPP3 | -1.01719 |
| GIPC1 | -1.01375 |
| ATP6V1F | -1.01282 |
| ATP5G1 | -1.01068 |
| SPINT2 | -1.01022 |
| SIGMAR1 | -1.00092 |
